# Spaser as a biological probe

**DOI:** 10.1038/ncomms15528

**Published:** 2017-06-08

**Authors:** Ekaterina I. Galanzha, Robert Weingold, Dmitry A. Nedosekin, Mustafa Sarimollaoglu, Jacqueline Nolan, Walter Harrington, Alexander S. Kuchyanov, Roman G. Parkhomenko, Fumiya Watanabe, Zeid Nima, Alexandru S. Biris, Alexander I. Plekhanov, Mark I. Stockman, Vladimir P. Zharov

**Affiliations:** 1 Arkansas Nanomedicine Center, University of Arkansas for Medical Sciences, Little Rock, Arkansas 72205, USA; 2 Institute of Automation and Electrometry of the Siberian Branch of the Russian Academy of Science, Koptyug Avenue 1, Novosibirsk 630090, Russia; 3 Nikolaev Institute of Inorganic Chemistry of the Siberian Branch of the Russian Academy of Science, Lavrentiev Avenue 3, Novosibirsk 630090, Russia; 4 Center for Integrative Nanotechnology Sciences, University of Arkansas at Little Rock, Little Rock, Arkansas 72204, USA; 5 Center for Nano-Optics and Department of Physics and Astronomy, Georgia State University, 29 Peachtree Center Avenue, Atlanta, Georgia 30302, USA

## Abstract

Understanding cell biology greatly benefits from the development of advanced diagnostic probes. Here we introduce a 22-nm spaser (plasmonic nanolaser) with the ability to serve as a super-bright, water-soluble, biocompatible probe capable of generating stimulated emission directly inside living cells and animal tissues. We have demonstrated a lasing regime associated with the formation of a dynamic vapour nanobubble around the spaser that leads to giant spasing with emission intensity and spectral width >100 times brighter and 30-fold narrower, respectively, than for quantum dots. The absorption losses in the spaser enhance its multifunctionality, allowing for nanobubble-amplified photothermal and photoacoustic imaging and therapy. Furthermore, the silica spaser surface has been covalently functionalized with folic acid for molecular targeting of cancer cells. All these properties make a nanobubble spaser a promising multimodal, super-contrast, ultrafast cellular probe with a single-pulse nanosecond excitation for a variety of *in vitro* and *in vivo* biomedical applications.

## Abstract (web summary)

Advanced diagnostic probes are required for monitoring disease progression. Here Galanzha *et al*. demonstrate a 22 nm plasmonic nanolaser to serve as a super-bright, biocompatible probe capable of generating stimulated emission directly inside living cells and animal tissue, while targeting cancer cells.

Detection of laser-excited spontaneous emission in fluorescence labels remains a powerful biological tool to study individual biomolecules and cells^1^,^2^,^3^. However, optical saturation at high laser intensity, photobleaching and blinking effects limit the detection sensitivity and practicality^4^. The lasing emission is resistant to saturation and photobleaching as in the case of the biolaser when biomolecules functioning as the gain medium are placed directly in the laser cavity^5^. However, this approach requires an external cavity exceeding cell size and limits possible biomolecule types yielding lasing. Stimulated emission is possible in the random laser using a turbid gain medium with no optical mirrors^6^,^7^; however, it requires relatively long optical pathways within the entire cell and high intracellular concentration of the gain medium, which limits medium types. Meanwhile, microresonator-based lasers are too large (≥4–10 μm) to be used for cellular labelling^8^,^9^. Plasmonic nanoparticles (NPs) are promising optical labels with low photothermal (PT) bleaching at realistic excitation levels as was demonstrated for PT and photoacoustic (PA) imaging and PA–PT theranostics^10^,^11^,^12^. However, the plasmonic NPs have wide absorption spectra and weak fluorescence, which limits their spectral selectivity and multimodal functionality, respectively. The sensitivity of methods using light scattering decreases dramatically for small NP sizes, which is especially detrimental in strong autofluorescence and scattering biological backgrounds^10^.

In contrast, the spaser (surface plasmon amplification by stimulated emission of radiation) consisting of a plasmonic NP as the optical resonator surrounded by a nanoshell of the gain medium can generate in a single mode emitting a spectrally tunable, bright light, fundamentally without a saturation. These properties are due to stimulated emission amplification effects^13^,^14^,^15^,^16^,^17^,^18^,^19^. However, biological applications of the spaser still remain elusive due to the challenges such as high optical losses, low solubility and toxicity. Here we introduce a biocompatible spaser that functions as one of the best multifunctional optical probes providing, simultaneously, super-bright stimulated emission, high PA imaging contrast and potential for PT destruction of cancer cells.

## Results

### Spasers

**Figure 1:**
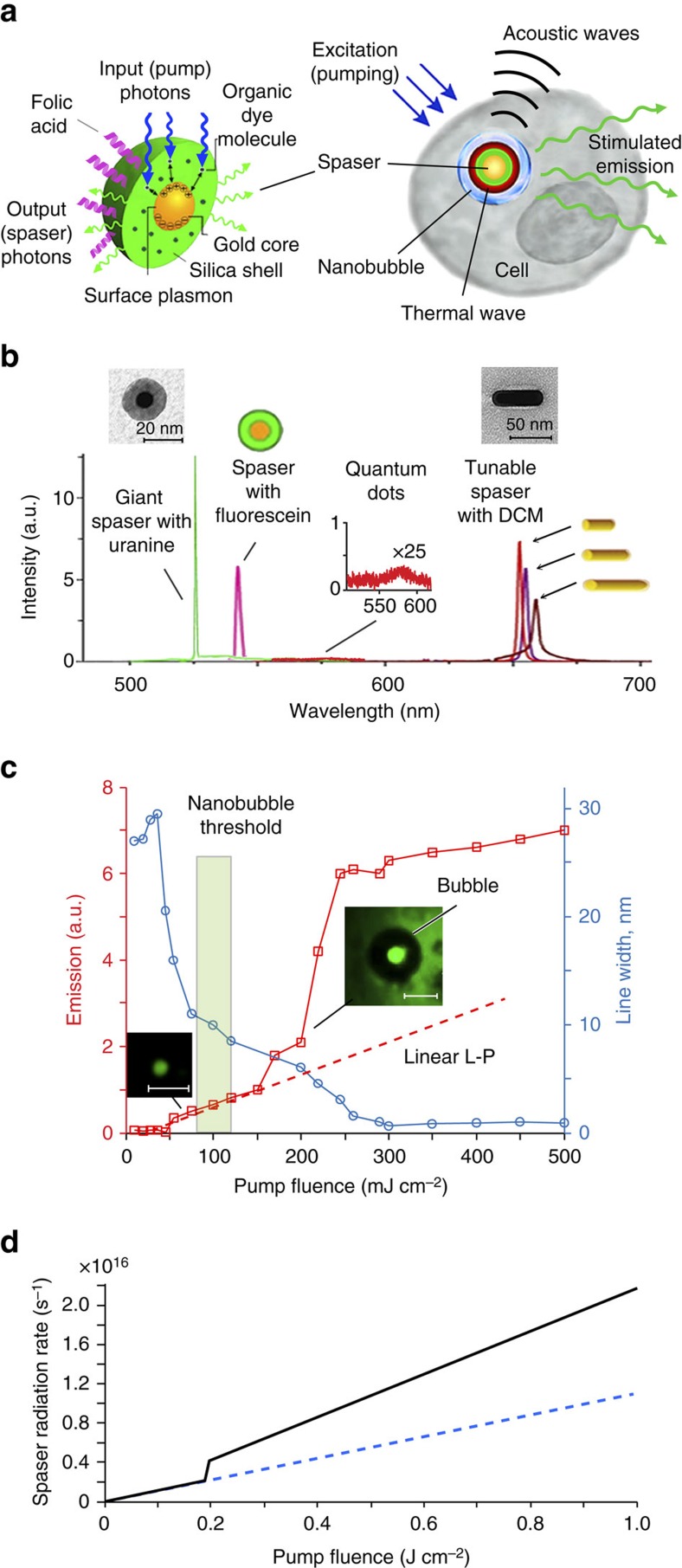
Study of spaser parameters. (**a**) Schematic of spaser as multimodal cellular nanoprobe. (**b**) Comparison of emission in suspensions of uranine spaser at 528 m (green peak; pump energy fluence 70 mJ cm^−2^), fluorescein spaser (magenta peak), QD with maximum at 576 nm (inset, red peak) and GNRs (right) with silica shell doped with DCM. TEM images shows 22-nm spaser (left inset) and GNRs (right inset). (**c**) Stimulated emission in spaser suspension. Red: input–output (light out–pump in (L–P)) curve (squares) of spasing. Blue: emission linewidth of the spasing. S.d. for intensity and linewidth are in the range of 18–26%. Scale bars 1 μm. (**d**) Theoretical L–L curve, that is, spaser photon radiation rate *I*_r_ versus pumping fluence *J*_p_ for spaser with a bubble (a 30 nm-thick vapour shell) forming at *J*_p_=0.2 J cm^−2^ (solid black curve). An L–L curve in the absence of the bubble formation is shown by a dashed blue line.

For biomedical applications, probes should be safe, water-soluble and super-bright in a biological background. We compared several spasers for their potential as biomedical probes. One type consisted of a 10-nm-diameter gold nanosphere core with a silica shell doped with various fluorescent dyes (Supplementary Note 1). Another type consisted of a gold nanorod (GNR) core with a silica shell doped with a DCM dye (4-(dicyanomethylene)-2-methyl-6-(4-dimethylaminostyryl)-4H-pyran) (Supplementary Note 2). The GNR-DCM spasers were prepared by incubation of DCM in ethanol solution in concentration of 0.3 mg ml^−1^ with GNRs in concentration of 10^12^ NPs cm^−3^. The structure and emission spectra of these spasers is illustrated in Fig. 1a,b. Comparison of spasers with the nanosphere cores and gain shells doped with uranine and fluorescein dyes demonstrated advantages of the uranine-based spasers as providing a stronger stimulated emission and a narrower emission line (1–2 nm versus 5–15 nm) and having a lower toxicity. Pump excitation of the spasers with the GNR cores was performed at a wavelength of 550 nm with a 7-ns pulse from an optical parametric oscillator (Solar LP601) focussed to a spot of 2 mm in diameter. The stimulated emission in GNR-DCM spasers was observed at wavelengths of 652, 654 and 660 nm depending on GNR length (60, 80 and 90 nm, respectively) at the same GNR diameter (20 nm) and the longitudinal surface plasmon resonances near 700, 766 and 808 nm, respectively (Fig. 1b). Emission of DCM alone centres at 638 nm. However, DCM is soluble in alcohols (ethanol or methanol) only, which are toxic for live cells. We explored also a planar lasing spaser (Supplementary Note 3, Fig. 1a–c) consisting of individual spasers with a 10-nm gold nanosphere core and a silica gain shell doped with fluorescein. These spasers were incorporated within the structure of artificial opal formed by 250 nm spherical silica particles, which were packed into an ordered face-centred cubic lattice structure forming 5 μm ‘photonic' films with a photonic band gap. Under pumping at a wavelength of 488 nm, spasing radiation has a pronounced six-fold symmetry of the reciprocal space (the first Brillouin zone). The threshold was 40 times lower and the amplification is much higher than for the same spasers in a suspension. However, the planar spaser is too large to serve as a cellular probe.

The main results of this research were obtained with a spaser consisting of a gold spherical NP (similar NPs are broadly used in the biomedical field)^10^,^11^,^12^ surrounded by a silica shell doped with a uranine (disodium fluorescein) dye (Fig. 1a, Supplementary Figs 2 and 3). This dye is widely used for tracing and biomedical diagnostics due to its low toxicity and high solubility in water and physiological solution^20^,^21^. Thus a favourable combination of properties of the chosen gold core, silica shell and uranine makes this spaser biologically safe and water soluble. This spaser is currently the smallest plasmonic nanolaser. Using advanced fluorescent, PT and PA techniques (Supplementary Fig. 4), we observed for a 22-nm spaser with a 10±1.9 nm NP diameter core and a 6±2.2 nm-thick shell (Fig. 1b, inset, left) the lasing threshold at a laser energy fluence of 26±6.3 mJ cm^−2^. Meanwhile, for a larger 60-nm spaser, the threshold was reduced down to 1.9±0.6 mJ cm^−2^ (Supplementary Fig. 5). Above the threshold, the light out–pump in (L–P) dependence demonstrated a characteristic straight line^14^,^15^ with emission spectral narrowing from 30–40 nm to 8–10 nm (Fig. 1c). Further increase of pump energy fluence led to formation of vapour nanobubbles around spasers. This phenomenon is primarily associated with laser heating of the spaser leading to evaporation of the liquid medium surrounding spaser (Supplementary Note 4)^11^,^22^,^23^. The nanobubble and microbubble formation was confirmed through specific imaging patterns (Fig. 1c inset, right) and nonlinear PA signal enhancement (Supplementary Fig. 6) as the bubbles act as an efficient PA signal amplifier^22^,^23^. The appearance of bubbles is accompanied by nonlinear enhancement of stimulated emission intensity and dramatic width narrowing (Fig. 1c). Specifically, irradiation of 60-nm diameter spaser with 10-nm-diameter gold core and ∼25-nm-radius silica shell in solution in a thin (≤0.5 μm) slide at energy fluence of 0.1 J cm^−2^ allowed us to observe the highest intensity of the stimulated emission, which we called ‘giant spasing'. The maximum ratio of the stimulated emission intensity to the spontaneous emission background was of 740±95 (Supplementary Fig. 7) The narrowest emission peak observed was of 0.8±0.2 nm. Both parameter levels were significant improvements over the previous results in factor of 10 and 6, respectively^14^,^15^,^16^,^17^,^18^,^19^.

### Nanobubble giant spasing

This unprecedented result of giant spasing can be explained by a theoretical model illustrated in Fig. 1d, which is based on general theory of spaser^24^. The details of this theory will be published elsewhere (Supplementary Note 4). It takes into account that the formation of the bubble leads to a reduction in the dielectric polarizability of the spaser. As a result, the frequency of the spasing plasmonic eigenmode increases and becomes closer to the transition in the gain medium (Supplementary Fig. 8). This leads to a significant increase of the number of plasmons in the spasing mode and, consequently, to an increase of the spaser radiation. The theoretical L–L (light out versus light in) curve in Fig. 1d agrees qualitatively very well with the experimental L–L curve (the red line in Fig. 1c). Nanobubble formation is also accompanied by ‘directional' emission (Supplementary Fig. 9) associated with scattering and refraction effects in nanobubbles.

Comparison of emission intensity between spaser and quantum dots (QDs), which are currently among the best conventional fluorescence probes^2^,^3^, revealed an ∼100-fold advantage in a higher brightness and up to 30-fold advantage in a narrower linewidth (0.8–1.5 nm versus 20–30 nm) for spasers at the same excitation parameters (Fig. 1b). While the maximum of spontaneous-radiation spectra was in the range of 535–540 nm, the spasing was observed in a blue-shifted range of 529±1 nm.

### Spaser as a molecular probe

**Figure 2:**
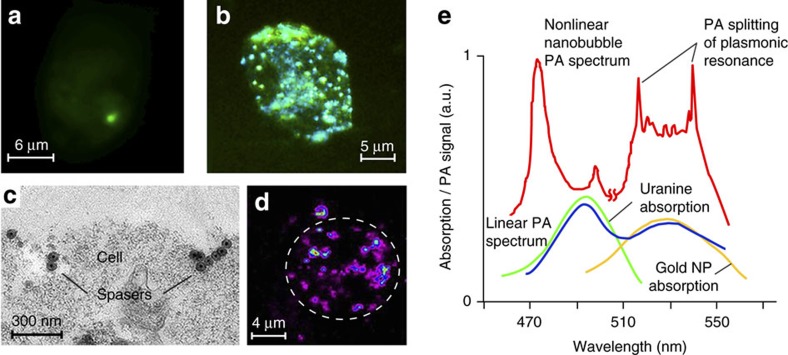
Spaser as multimodal contrast agent. (**a**,**b**) Fluorescence image of breast cancer cells (MDA-MB-231) with a single spaser (**a**) and multiple spasers (**b**). Pump parameters: wavelength 488 nm; pump pulse width 10 ns; beam diameter 20 μm; energy fluence 80 mJ cm^−2^ (**a**) 150 mJ cm^−2^ (**b**). Incubation time: 10 min (**a**) and 60 min (**b**). (**c**) TEM image of single and clustered spasers on a breast cancer cell (MDA-MB-231) membrane after 30 min cell incubation at 37 °C; (**d**) PT image of cancer cell labelled with spasers (false colours). Labelling parameters: incubation time, 1 h; temperature, 37 °C; spaser concentration, ∼10^12^ cm^−3^. Laser parameters; wavelength, 532 nm; pulse width, 5 ns; pulse rate, 100 Hz; energy fluence, 20 mJ cm^−2^. (**e**) Absorption spectra of uranine (green) and gold NPs (yellow) and normalized PA spectra of spasers in linear (blue) and nonlinear (red) modes at energy fluences of 50 and 110 mJ cm^−2^, respectively.

To validate the functionality of the spaser in a biological environment, the spasers have been conjugated with a low-molecular-weight folic acid to target the folate receptor, which is commonly overexpressed on the surface of most human cancer cells and is relatively weakly expressed in normal cells^22^,^25^. The presence of folic acid on spaser's surface was confirmed through appearance of folate-specific spectral peaks in absorption and Raman spectra of spasers (Supplementary Fig. 10a,b)^25^. To verify the targeting specificity, the conjugated and non-conjugated spasers in a concentration of 10^12^ NPs cm^−3^ were incubated for different times (5, 30 and 60 min) with breast cancer cells (MDA-MB-231) and endothelial cells (2H11) with a high and low folate receptor expression, respectively. Cells with the spasers demonstrated high image contrasts exhibiting one (Fig. 2a) or many individual ‘hot spots' (Fig. 2b) at different laser energies above the spasing threshold. The number of these spots increases with both increasing laser energy and incubation time.

The following data suggest that the observed discrete image structure resulted from spatially isolated single spasers. First, the presence of spasers inside cells was confirmed with transmission electron microscopy (TEM; Fig. 2c), inductively coupled plasma mass spectrometry (ICP-MS) (Supplementary Note 5), scanning transmission electron microscopy (STEM) (Supplementary Note 6, Supplementary Figs 2b and 11), energy-dispersive X-ray spectroscopy (EDX) with STEM imaging, PT microscopy (Fig. 2d, Supplementary Fig. 4) and PA flow cytometry using strongly absorbing gold core as high PT and PA contrast agent (for details of these techniques, see refs 11, 22, 23). These techniques revealed an initial accumulation of individual spasers on the cell membrane (Fig. 2c, Supplementary Fig. 11) followed by their internalization in the cell cytoplasm with spaser quantities varying from 1–5 to several hundred with an increasing incubation time. The STEM images showed that the functionalization did not result in aggregation of spasers but rather primarily in closely located single spasers (spaser clusters). The spaser density was consistent with the value that was estimated from the sample volume and concentration. Although the spaser emission in solution and in the cells varied significantly, up to 30–40% due to an inhomogeneous dye concentration, differences by 2–3 times, which can be associated with spaser's clusters, were very rare.

Linear PA spectroscopy at a relatively low laser energy (Fig. 2e, bottom) was used for spectral spaser identification through appearance of two characteristic maxima in the PA spectra at 490 and 530 nm associated with dominant uranine and gold NP absorption, respectively. Nonlinear nanobubble-related mode led to splitting of plasmonic peak in two blue- and red-shifted sharp peaks (Fig. 2e, top). This phenomenon is associated with spectral burning of the centre of absorption bands producing central spectral hole, while lower absorption outside the absorption centre still provides generation of nanobubbles as a PA signal's amplifier on wings of absorption band^11^,^26^. This leads to the aforementioned blue and red sharp peaks. Recently, these peaks were observed in infrared range by an independent research group^26^.

A high speed (1,000 cells per minute) PA flow cytometry^22^,^23^ demonstrated a high labelling efficiency through significant (10–50-fold) signal increase from individual cells loaded with spasers (Supplementary Fig. 12). Conjugated spasers bound to 37±3.1%, 76±3.3% and 89±3.9% of the cancer cells during 10, 30 and 60 min incubation, respectively (Table 1). In contrast, nonspecific binding of conjugated spasers to endothelial cells was in the range of 5–11%, while nonspecific binding of non-conjugated spasers was almost negligible (≤0.5–2%) during a short time (10–30 min).

**Figure 3:**
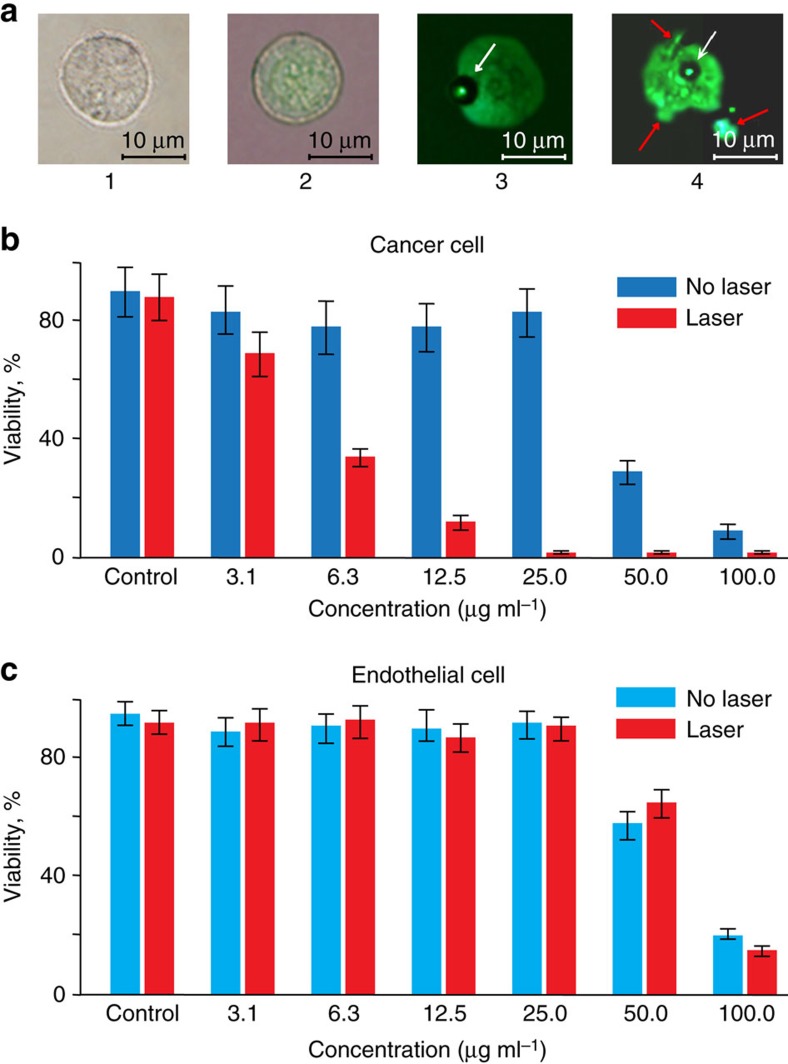
Spaser as an efficient biocompatible theranostic agent. (**a**) Bright field image of control cancer cell (MDA-MB-231) with no spasers (1), combined fluorescent and bright field image of the same cell labelled with spasers at a low concentration (2); fluorescent image of bubble formation (3, arrow) in a cell with a moderate spaser concentration (3); bubble formation (white arrow) leading to cell fragmentation (red arrows) at a high spaser concentration (4). (**b**) Cell viability tests for cancer cells (MDA-MB-231) at different spaser concentration using CellTiter-Glot assay before (blue) and after (red) laser irradiation (100 mJ cm^−2^, 1 Hz, 3 min). (**c**) Cell viability tests for endothelial cells (2H11) at different spaser concentrations using CellTiter-Glot assay before (blue) and after (red) laser irradiation (100 mJ cm^−2^, 1 Hz, 3 min). S.d. for each column are in the range of 15–20%.

Important for biological application, cytotoxicity of the spasers was estimated at different concentrations and incubation times for the cancer and endothelial cells before and after laser irradiation. We found low toxicity for both cell lines with viability above 92–96% in a broad concentration range up to 1–50 μg ml^−1^, which is consistent with safety of uranine (refs 20, 21). Laser irradiation of the cancer and endothelial cells incubated with the spasers led to decreased cell viability with an increased spaser concentration with no notable viability changes for control cells with no spasers (Fig. 3b,c, Supplementary Fig. 13). Comparison of images of cells at different laser energies (Fig. 3a) and spaser concentrations revealed that mechanism of cell damage was associated with laser-induced vapour nanobubbles around overheated spasers, especially their clusters leading initially to mechanical damage of the cell membrane and then to complete cell fragmentation. These data suggest a high potential of spasers as theranostic agents integrating optical diagnosis and PT-based cell killing using just a few laser pulses (Supplementary Fig. 13).

### Spasing *in vivo*

**Figure 4:**
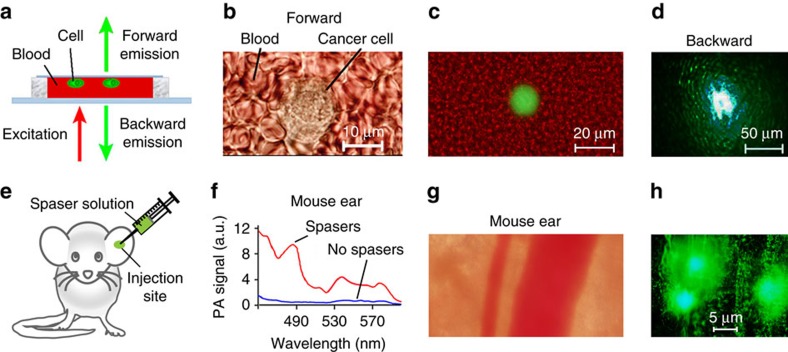
Detection of spasers and labelled cancer cells *in vitro* and in animal model (mouse) *in vivo.* (**a**) Schematic of laser irradiation of cells in blood in slide. (**b**,**c**) Transmission (**b**) and combined fluorescence and transmission (**c**) image at energy fluence of 20 mJ cm^−2^ (below the spasing threshold) of single cancer cell in human blood in slide in forward direction. (**d**) Fluorescence images of single cell in human blood at depth of 1 mm in backward direction at energy fluence of 120 mJ cm^−2^ (above the spasing threshold). (**e**) Schematic of intradermal injection of spaser suspension into of mouse ear tissue. (**f**) PA spectral identification of spasers in ear tissue inside (red) and outside (blue) injection area using tunable optical parametric oscillator (OPO) with diameter of 15 μm and energy fluence of 20 mJ cm^−2^. (**g**,**h**) Transmission (**g**) and fluorescence (**h**) images of mouse ear fragment with blood vessels and two spots with spasers. Laser pump parameters: wavelength, 488 nm; beam diameter: 50 μm; energy fluence intensity, 120 mJ cm^−2^.

To simulate spasers' optical signatures, we recorded emission from cells labelled with the spasers through a layer of human blood (Fig. 4a–c). We were able to detect emission through a ∼1 mm-thick blood layer (Fig. 4d), which would have been impossible for QDs where a typical observation depth is ≤100 μm in visible range. *In vivo*, similar results were demonstrated by local injection of spasers in a mouse ear tissue (Fig. 4e). The PA spectroscopy was used for additional verification of the presence of the spasers in the injected site through monitoring of increased PA signal amplitudes (Fig. 4f) and through spectra typical for the spasers. Pump irradiation caused strong emissions from the ear tissue (Fig. 4g) with a high local spaser concentration (Fig. 4h).

## Discussion

We have demonstrated versatile functionalities of the spaser in complex biological backgrounds from cellular cytoplasm *in vitro* to a mouse tissue *in vivo*. The spasers excited by just a single nanosecond laser pulse served as low-toxicity ultrafast probes, with a molecular specificity, the narrowest emission spectra (∼1 nm) and the highest brightness emission. This is impossible to achieve with conventional QD and even advanced NP-based fluorophores^3^. Compared to spontaneous emission from the fluorescent dyes alone, the stimulated emission in spasers with the same dye in the gain medium is much higher at the same excitation energy before undergoing photobleaching (Supplementary Note 7, Supplementary Fig. 14). This is due to the fact that the spaser emission does not saturate, and the spasers are more resistant to photobleaching. The demonstrated spasers with the sizes of just 22 nm set current record for the smallest nanolasers, which are ∼100-fold smaller (in linear size) than microlasers^8^ and the biolaser using liquid microdroplets as optical resonators^27^. Moreover, we have showed multimodal capability of the spasers as light-emitting, PT- and PA-contrast agents for diagnosis and spectral identification in complex absorption backgrounds through appearance of spaser's spectral fingerprints, especially, PA peak's splitting (Fig. 2e). Moreover, just a few laser pulses are sufficient for killing cancer cells using combination of PA diagnosis and PT therapy (PA–PT spaser theranostics). There is a clearly defined and promising field for spaser-based therapeutic applications and high-contrast imaging with low photobleaching *in vitro* and *in vivo* at a single-cell level using energy fluence levels that are close to the laser safety standards^28^. Note that potential dye photobleaching and spaser degradation and at higher fluences is not at all important in single-pulse theranostic applications. Further developments may encompass extension of the spectral range from visible using gold spherical cores^14^ and their cluster^29^,^30^ to near-infrared tissue-transparency window (750–1,100 nm) with GNR cores^17^,^31^, making advantage of the narrow spaser emission and ultrasharp PA resonances (Fig. 2e)^11^,^26^ for further increase of the detection sensitivity and specificity. It can be achieved by narrow-band spectral filtering and potential spectral tunability using various spaser types (Fig. 1b, Supplementary Fig. 1)^17^,^18^ for ultrasharp colour multiplexing without spectral overlap and functionality providing by conjugation with various ligands (for example, folate or antibodies).

## Methods

### Synthesis of spacers

Gold NPs were prepared by the standard method^32^. The next step was the modification of the gold surface by the addition of a freshly prepared aqueous solution of (3-aminopropyl) trimethoxysilane (5 μl, 1 mM) to the colloid under magnetic stirring. The NPs were then individually coated by a sodium silicate shell by the addition of active silica (3 ml, 0.54 weight% sodium silicate solution) with pH∼10. After 1 day, NPs were transferred into ethanol (10 ml sol: 40 ml ethanol). The well-known Stober method was used to grow the shells. The obtained colloid was mixed with the physiological solution of uranine C_20_H_10_O_5_Na_2_ (2 × 10^−2^ M or 7.5 mg ml^−1^) in equal volumes. Then the colloid was allowed to stand for 2 days to allow the infiltration of the dye into the shells. The concentration of the gold-shell NPs in a colloid was ∼2 × 10^12^ cm^−3^, calculated from the gold weight fraction measurements. Spasers were functionalized with folate using the established procedure^25^. Folic acid (>97%) obtained from Sigma-Aldrich Corp. was used without additional purification. Best conjugation was achieved by covalent conjugation of folate to silica shells following dye loading. A small blue shift of the bonds corresponding to folic acid in the Raman spectra compared to pure folic acid can be ascribed to the interaction of the folic acid with silica surface (Supplementary Fig. 10). The obtained Au-SiO_2_ complex was centrifuged at 10,000 r.p.m. for 30 min. Supernatant was removed and solid was re-dispersed in 20 ml anhydrous ethanol. Subsequently, 5 ml APTS was added to mixed solution and allowed to react at 40 °C for 48 h for obtaining Au-SiO_2_-NH_2_ structure. The resultant was washed with ethanol once and by deionized water two times and re-dispersed in 10 ml ethanol. Binding of folic acid to the Au-SiO_2_-NH_2_ was carried out using a modification of the standard EDC–NHS reaction. Carboxyl groups of folic acid were activated by an EDC/NHS solution for 20 min. Following activation, 3 mg Au-SiO_2_-NH_2_ were added to form a mixed solution and allowed to react at room temperature for 24 h. The degree of binding of folic acid and amine groups depends on many factors: pH, amine/carboxyl ratio, EDC/NHS ratio, and duration of reaction. Under our conditions, the degree of binding of folic acid and amine groups was ≥50%. The resultants were washed by distilled water three times for removing unreacted chemicals by centrifugation. For better purification, we increased the number of centrifugation procedure. Furthermore, the last supernatant was investigated using ultraviolet–visible and Raman spectroscopy, and the characteristic peaks of folic acid were not observed in the spectra. Therefore, we can confirm that free folic acid in the solution of spasers is absent.

### Experimental PT and PA setups

Spaser characterization was performed with a multimodal PT and PA techniques described elsewhere^22^,^23^ (Supplementary Fig. 4) Briefly, the PT microscopic module was built on technical platform of an Olympus IX81 inverted microscope (Olympus America, Inc.) using a tunable optical parametric oscillator (OPO; Opolette HR 355 LD, Opotek, Inc.) with the following parameters: tunable spectral range 420–2,200 nm; pulse width 5 ns; pulse repetition rate 100 Hz, and fluence range 0.1–10^4^mJ cm^−2^. Laser beams were focussed into the sample with various magnification objectives, including × 100 oil-immersion objective (DPlan 100, NA 1.25, Olympus, Inc.) and × 40 (Ach, NA 0.65, Olympus, Inc.). In a PT thermal lens mode, pump (OPO) laser-induced temperature-dependent variations of the refractive index caused a collinear He-Ne laser probe beam (model 117A, Spectra-Physics, Inc.; wavelength 633 nm; power 1.4 mW) to defocus and, hence, a reduction in the beam's intensity at its centre. The probe laser light was collected after the sample using either a × 100 water-immersion (LUMPlanFl 100, NA 1.00, Olympus, Inc.) or a × 40 objective (Ph3 DL, NA 0.55, Nikon Inc) and was detected by a photodiode (PDA36A, Thorlabs, Inc) with a 50-μm pinhole (referred to as PT signals). The PT signals demonstrate an initial peak associated with rapid, picosecond-scale heating of dyes or NPs and with a slower, nanosecond-to-microsecond-scale, exponential tail corresponding to target cooling. In the nonlinear mode, laser-induced nanobubbles around overheating strongly absorbing NPs lead to the appearance of sharp negative peaks associated with refraction and scattering of the probe beam on the nanobubbles (Supplementary Fig. 4b, inset). In the confocal scheme, the plane of the pinhole is fixed at one Rayleigh distance from the probe-beam waist. The PT images are constructed by acquiring the PT signals from a sample as it undergoes scanning in *x*–*y* dimensions using a two-dimensional stage (H117 ProScan II, Prior Scientific, Inc.) with the scanning step of 0.25–1 μm. The PT signals were recorded using a 200-MHz analogue-to-digital converter board (PCI-5152, National Instruments Corp.) and analysed by custom software (LabVIEW 8.5, National Instruments Corp.). A Dell Precision 690 workstation provided signal acquisition and processing, synchronization of the excitation laser and translation-stage control. Resolution is determined by the microscope objective itself (for example, ∼0.7 μm at × 20, NA 0.4; and ∼250 nm at × 100, NA 1.25).

In the PA module, the PA signals from an ultrasound transducer (unfocussed XMS-310 with a 10-MHz frequency band or focussed V316-SM with a 20-MHz frequency band; both from Panametrics) and an amplifier (model 5,662 or 5,678, Panametrics) were recorded using customized software. Fluorescence imaging, added to verify PT and PA data, was performed with a cooled colour CCD camera (DP72, Olympus). Navigation of the laser beams for the *in vitro* study was controlled with a high-resolution (∼300 nm) transmission digital microscopy module.

Labelling efficiency of cancer and endothelial cells by spasers was estimated with PA flow cytometry integrated with fluorescent flow cytometry^33^. Briefly, system was built on a microscope platform and customized high-pulse-repetition-rate nanosecond and continuous wave lasers with the following parameters: (1) wavelength 532 nm; pulse width 8 ns; pulse rate 10 kHz; pulse energy 10 μJ; and (2) continuous wave diode laser with a wavelength of 488 nm and power of 50 mW (IQ1C45(488–60)G26, Power Tech., Alexander, AR). Laser beams were delivered to the flow system through a customized optical system with a combination of spherical and cylindrical lenses forming a linear beam with size of 5 μm × 2 mm on a quartz capillary with the size of 200 μm × 2 mm and flow rate of 1 ml min^−1^. The PA signals were acquired by an unfocussed ultrasound transducer (2.25 MHz, V-323-SM; Olympus-NTD, Waltham, MA, US) and further amplified (for example, by preamplifier 5662B, Panametrics), digitized (custom AD484, high-speed digitizer, 4DSP, Inc.), recorded and analysed by customized software. A × 40 micro-objective (NA 0.65; Olympus) was used for collection of fluorescence light from cells.

### Other analytical techniques

Thermogravimetric analysis (TGA) was used for estimation of dye content in spaser shell through its changes in physical and chemical properties of materials that are measured as a function of increasing temperature. The TGA was performed at atmospheric pressure in helium flow (30–40 ml min^−1^) with a heating rate of 1 °C min^−1^ within the temperature range of 25–900 °C using Thermo-Microbalance TG 209 F1 Iris. A standard open crucible was used. After centrifugation, the suspension of spasers was dried and then was a subject of thermogravimetric test. The TGA showed that after heating up to 900 °C a residual mass of hybrid NPs (spasers) containing or not containing the dye in the shell was 62.5% (or 0.57 mg) due to decomposition of the dye and complete evaporation of the pore water in the shells. At a temperature of *T*_crit_ ∼500 °C, which corresponded to the decomposition temperature of the dye, the difference of their weight was about 3% or 0.028 mg, which corresponded to the total number of dye molecules *N*_dye_=2.8 × 10^−5^ × 6 × 10^23^/376=4.5 × 10^16^ molecules. Based on the evaluation of a mass of NPs with a diameter of 60 nm, *m*_NP_=2.5 × 10^−16^ g, the number estimated to be 0.57 × 10^−3^/2.4 × 10^−16^=2.3 × 10^12^ NPs, that is, the shell contained about 1.9 × 10^4^ dye molecules. Our measurements also showed that the amount of dye in the shell per one gold NP was about 2.7 × 10^3^ dye molecules for 30 nm NPs.

In order to quantify the uptake of spasers by cells, several analytical methods were used (Supplementary Notes 5 and 6, Figs 2 and 4 and Supplementary Figs 10–12): ICP-MS, TEM, EDX, and STEM. TEM and STEM images were collected by JEOL JEM 2100F (JEOL USA, Peabody, MA) equipped with an EDAX Genesis (Ametek, Berwyn, PA) EDS X-ray analyser. Both TEM bright-field images and STEM high-angle annular dark-field images were generated at 80 kV bias, and STEM images are collected with the probe size of 1.5 nm and the camera length 20 cm. EDAX Genesis EDS system was also used to collect X-ray spectra from the specimen. A few drops of sample solution were deposited on holey carbon-coated copper TEM grid (SPI Supplies, West Chester, PA) and dried before the grid was entered into the TEM.

In addition, ultraviolet–visible spectrophotometry (Shimadzu 2,500 ultraviolet–visible) and Raman techniques (SPEX Triplemate 1877) were used for spectroscopic spaser evaluation, in particular, to confirm conjugation of the spasers with folate through appearance of folate-related spectral peaks in absorption and Raman spectra of the spasers after removal of free folic acid in solution during extensive washing (Supplementary Fig. 10).

### Study of spaser emission

To study the stimulated emission, samples (for example, spaser solution) were loaded either in a cuvette (with oblique walls to eliminate feedback) of 2-mm path length, and microscopic slides of 120 and 1-μm path lengths. The samples were irradiated by OPO (Solar LP601 and above) at wavelength *λ*=488 nm with 5–7-ns pulses focussed into spots of different diameters from 1 to 100 μm. For spectroscopic measurements, we used a fibre-optic-based spectrometer (OceanOptics USB4000) with optical resolution of Δ*λ*∼0.1 nm (full-width at half-maximum) or AvaSpec-2048 TEC-FT-2 (Δ*λ*∼0.7 nm (full-width at half-maximum)) (Supplementary Fig. 4c).

### Cells

Human breast cancer cells (MDA-MB-231, American Type Culture Collection (ATCC)) with high folate receptor expression and endothelial cells (2H11, ATTC CRL-2163) with low folate receptor expression were used as targeted and control cells. Both types of cells were cultured according to vendors' specifications. In particular, cancer cells were maintained in an α-minimum essential medium (Invitrogen/Life Technologies) with 5% fetal bovine serum and penicillin–streptomycin (Invitrogen/Life Technologies). Viable non-contaminated cells (cells were tested for mycoplasma contamination) were resuspended in PBS at a concentration of ∼1 × 10^6^ cells per 1 ml and labelled with spasers at different incubation times (10, 30 and 60 min, at 37 °C).

### Animal and human study

According to University of Arkansas for Medical Sciences Institutional Animal Care and Use Committee approved protocols, the experiment involved nude female mice (*nu/nu*) aged 5–6 weeks purchased from a commercial source. The animals were anaesthetized by isoflurane and placed on a microscopic stage. Ultrasound gel was used for acoustic matching of the ultrasound transducer and the ear tissue. The positions of ear structures, pump beam and injected site were visualized by optical and fluorescence imaging.

Fresh blood was obtained from healthy donors in heparinized tubes in accordance with protocols approved by the UAMS Institutional Review Board. Informed consent was obtained from the human subjects.

### Study of cell viability

The impact of the laser irradiation on live cells in the presence of the spasers was estimated with standard cell viability assays: trypan blue and CelTiter-Glo kits according to the manufacturer's procedures. The highest spaser concentration in the suspension (Fig. 3) was sequentially diluted several times.

### Statistical analysis

Results are expressed as means±s.d. and the confidence interval of at least three independent experiments (*P* > 0.95). Statistica 5.11 (StatSoft, Inc.), MATLAB 7.0.1 (MathWorks) and LabVIEW (National Instruments) were used for the statistical calculations. Data were summarized as the mean, s.d., relative s.d., median and full range. The error bars in figures represent s.d. In the selected figures, we only indicated the average s.d. in the caption to provide more clear data presentation.

### Data availability

The authors declare that relevant data supporting the findings of this study are available on request.

## Additional information

**How to cite this article:** Galanzha, E. I. *et al*. Spaser as a biological probe. *Nat. Commun.* **8**, 15528 doi: 10.1038/ncomms15528 (2017).

**Publisher's note:** Springer Nature remains neutral with regard to jurisdictional claims in published maps and institutional affiliations.

## Supplementary Material

Supplementary Information Supplementary Figures, Supplementary Notes, Supplementary Table and Supplementary References

## Figures and Tables

**Table 1:** Labelling efficiency (%) of breast cancer cells (MDA-MB-231) and endothelial cells (2H11) obtained with a PA flow cytometry.

**Cells**	**Spasers**	**Incubation time (min)**		
		**10**	**30**	**60**
Cancer	Conjugated	37	76	89
	Non-conjugated	1	2.1	11
Endothelial	Conjugated	1	4.5	8
	Non-conjugated	—	2	7

## References

[b1] Resch-Genger U., Grabolle M., Cavaliere-Jaricot S., Nitschke R. & Nann T. Quantum dots versus organic dyes as fluorescent labels. Nat. Methods 5, 763–775 (2008). 18756197 10.1038/nmeth.1248

[b2] Bruchez M., Moronne M., Gin P., Weiss S. & Alivisatos A. P. Semiconductor nanocrystals as fluorescent biological labels. Science 281, 2013–2016 (1998). 9748157 10.1126/science.281.5385.2013

[b3] Chan Y.-H. . Hybrid semiconducting polymer dot-quantum dot with narrow-band emission, near-infrared fluorescence, and high brightness. J. Am. Chem. Soc. 134, 7309–7312 (2012). 22515545 10.1021/ja3022973 PMC3350096

[b4] Letokhov V. Laser biology and medicine. Nature 316, 325–330 (1985). 3894977 10.1038/316325a0

[b5] Fan X. & Yun S. The potential of optofluidic biolasers. Nat. Methods 11, 141–147 (2014). 24481219 10.1038/nmeth.2805 PMC4162132

[b6] Letokhov V. S. Generation of light by a scattering medium with negative resonance absorption. Sov. J. Exp. Theor. Phys. 26, 835–840 (1968).

[b7] Polson R. & Vardeny Z. Random lasing in human tissues. Appl. Phys. Lett. 85, 1289–1291 (2004).

[b8] Humar M. & Yun S. Intracellular microlasers. Nat. Photon. 9, 572–576 (2015). 10.1038/nphoton.2015.129 PMC4583142 26417383

[b9] Schubert M. . Lasing within live cells containing intracellular optical microresonators for barcode-type cell tagging and tracking. Nano Lett. 15, 5647–5652 (2015). 26186167 10.1021/acs.nanolett.5b02491

[b10] Boyer D., Tamarat P., Maali A., Lounis B. & Orrit M. Photothermal imaging of nanometer-sized metal particles among scatterers. Science 297, 1160–1163 (2002). 12183624 10.1126/science.1073765

[b11] Zharov V. P. Ultrasharp nonlinear photothermal and photoacoustic resonances and holes beyond the spectral limit. Nat. Photon. 5, 110–116 (2011). 10.1038/nphoton.2010.280 PMC4282491 25558274

[b12] Wang L. V. & Hu S. Photoacoustic tomography: *in vivo* imaging from organelles to organs. Science 335, 1458–1462 (2012). 22442475 10.1126/science.1216210 PMC3322413

[b13] Bergman D. J. & Stockman M. I. Surface plasmon amplification by stimulated emission of radiation: quantum generation of coherent surface plasmons in nanosystems. Phys. Rev. Lett. 90, 027402 (2003). 12570577 10.1103/PhysRevLett.90.027402

[b14] Noginov M. A. . Demonstration of a spaser-based nanolaser. Nature 460, 1110–1112 (2009). 19684572 10.1038/nature08318

[b15] Stockman M. Nanoplasmonics: the physics behind the applications. Phys. Today 64, 39–44 (2011).

[b16] Lu Y. J. . Plasmonic nanolaser using epitaxially grown silver film. Science 337, 450–453 (2012). 22837524 10.1126/science.1223504

[b17] Meng X., Kildishev A. V., Fujita K., Tanaka K. & Shalaev V. M. Wavelength-tunable spasing in the visible. Nano Lett. 13, 4106–4112 (2013). 23915034 10.1021/nl4015827

[b18] Lu Y. J. . All-color plasmonic nanolasers with ultralow thresholds: autotuning mechanism for single-mode lasing. Nano Lett. 14, 4381–4388 (2014). 25029207 10.1021/nl501273u

[b19] Ma R. M., Ota S., Li Y., Yang S. & Zhang X. Explosives detection in a lasing plasmon nanocavity. Nat. Nanotechnol. 9, 600–604 (2014). 25038780 10.1038/nnano.2014.135

[b20] Richard G., Soubrane G. & Yanuzzi L. Fluorescein and ICG Angiography: textbook and Atlas Thieme Medical Publishers (1998).

[b21] Alford R. . Toxicity of organic fluorophores used in molecular imaging: literature review. Mol. Imaging 8, 341–354 (2009). 20003892

[b22] Galanzha E. I. . *In vivo* magnetic enrichment and multiplex photoacoustic detection of circulating tumour cells. Nat. Nanotechnol. 4, 855–860 (2009). 19915570 10.1038/nnano.2009.333 PMC3663137

[b23] Sarimollaoglu M., Nedosekin D. A., Menyaev Y. A., Juratli M. A. & Zharov V. P. Nonlinear photoacoustic signal amplification from single targets in absorption background. Photoacoustics 2, 1–11 (2014). 24921062 10.1016/j.pacs.2013.11.002 PMC4048727

[b24] Stockman M. I. The spaser as a nanoscale quantum generator and ultrafast amplifier. J. Opt. 12, 024004-024001-024013 (2010).

[b25] Huang P. . Folic acid-conjugated silica-modified gold nanorods for X-ray/CT imaging-guided dual-mode radiation and photo-thermal therapy. Biomaterials 32, 9796–9809 (2011). 21917309 10.1016/j.biomaterials.2011.08.086

[b26] Mertiri A. . Nonlinear midinfrared photothermal spectroscopy using Zharov splitting and quantum cascade lasers. ACS Photon. 1, 696–702 (2014). 10.1021/ph500114h PMC4270413 25541620

[b27] Jonas A. . *In vitro* and *in vivo* biolasing of fluorescent proteins suspended in liquid microdroplet cavities. Lab Chip 14, 3093–3100 (2014). 24968888 10.1039/c4lc00485j

[b28] American National Standard for Safe Use of Lasers ANSI Z136.1. (2007).

[b29] Parkhomenko R. G. . Gold nanostructure formation in the photonic crystal matrix by means of MOCVD technique. Surf. Coat. Technol. 230, 279–283 (2013).

[b30] Kuchaynov A. S. . in *The Seventh International Conference on Material Technologies and Modeling MMT**-2012*, 67–71 (Ariel, Israel, 2012).

[b31] Benimetskiy F., Plekhanov A., Kuchyanov A., Parkhomenko R. & Basova T. in *Days on Diffraction* **2016**, 62–66 (St. Petersburg, Russia, 2016).

[b32] Liz-Marzan L. M., Giersig M. & Mulvaney P. Synthesis of nanosized gold-silica core-shell particles. Langmuir 12, 4329–4335 (1996).

[b33] Cai C. . *In viv*o photoacoustic flow cytometry for early malaria diagnosis. Cytometry A 89, 531–542 (2016). 27078044 10.1002/cyto.a.22854

