# Supplementary material for: Spaser as a biological probe
Source: Nat Commun. 2017 Jun 8;8:15528. doi: 10.1038/ncomms15528 (PMC5472166; doi:10.1038/ncomms15528)
Supplement: Supplementary Information — Supplementary Figures, Supplementary Notes, Supplementary Table and Supplementary References [file ncomms15528-s1.pdf]

# Supplementary information

## Spaser as a biological probe

**Supplementary Note 1 | Spaser with gold nanosphere core and fluorescein dye**

We explored spasers[1-4] consisting of 10-nm-diameter gold core and a 6-nm silica shell doped with fluorescein. To study the stimulated emission, spasers in suspension were put in a cuvette (with oblique walls to eliminate feedback) of 2 mm path length and pumped at wavelength $\lambda$ = 488 nm with 7-ns pulses from an optical parametric oscillator (Solar LP601) slightly focused into a 1-mm spot[2]. With increasing pump pulse energy, an emission peak appeared initially at 552 nm and narrowed to 15-18 nm, then shifted to 540 nm once the pumping energy exceeded a critical threshold value. In analogy with priory work (e.g., see refs. 1-4 and cited there), sequentially diluting the sample decreased the emission intensity, but did not change the character of the spectral line or diminish the ratio of stimulated emission intensity to spontaneous emission background. This suggests that the observed stimulated emission has been produced by individual spasers. Also, the emission dynamics detected at 540 nm showed shortening of the emission pulse when the pumping energy exceeded a lasing threshold. This is an additional proof of the spasing occurring in individual spasers. Comparison of spasers doped with uranine and fluorescein dyes demonstrated the advantages of uranine-based spasers in more profound stimulated emission and more narrow emission line at lower toxicity.

**Supplementary Note 2 | Spasers with gold nanorod core and DCM dye**

We explored spaser with a gold nanorod (GNR) core[1,2] and silica layer doped with a dye: DCM [4-(Dicyanomethylene)-2-methyl-6-(4-dimethylaminostyryl)-4H-pyran]. The spasers were prepared by incubation of DCM in ethanol solution at a concentration of 0.3 mg ml$^{-1}$ with GNRs at a concentration of $10^{12}$ cm$^{-3}$. Pump excitation was performed at a wavelength of 550 nm with a 7-ns pulse from an optical parametric oscillator (Solar LP601) focused to a 2-mm spot. Stimulated emission in the GNR-DCM spaser suspension was observed at wavelength near 652 nm, 654 nm, and 660 nm depending on GNR length (60 nm, 80 nm, and 90 nm, respectively) at the same diameter (20 nm) and the longitudinal surface plasmon resonances were observed near 700, 766, and 808 nm, respectively (Fig. 1B). Emission of DCM alone occurs at 638 nm. Stimulated emission width peak was 3-5 nm at pump intensity above the threshold of a 3 MW cm$^{-2}$. However, the DCM is soluble in ethanol (or methanol) only, which is toxic for live cells. This prevents biomedical application of this dye solution for live tissue.

**Supplementary Note 3 | Film-based spasers using clustered nanospherical core and fluorescein dye**

We explored a planar lasing spaser (Supplementary Fig. 1A) consisting of a 10-nm gold nanosphere core with silicate doped with fluorescein (Note 1)[3,4]. These spasers were incorporated within the structure of artificial opal formed from ~250 nm spherical silica particles, which are packed into an ordered face-centered cubic lattice structure forming a 5 μm "photonic" film with a photonic band gap (Supplementary Fig. 1B). The diameter

of the silica spheres is chosen so that the Bragg reflection wavelength of this film overlaps with the optical-gain spectrum of the light-emitting gold core providing necessary optical feedback. In this schematic, the direction of emission is normal to the plane of the optical surface, where the plasmonic resonators oscillate in phase. The film with the spasers was pumped at a wavelength of $\lambda$ = 488 nm with a 7-ns pulse from an optical parametric oscillator (Solar LP601) focused to a 2-mm spot in diameter. At the low pump energy, only luminescence was observed with maximum near 552 nm. As the pump energy increased above the threshold 140 kW $cm^{-2}$ (0.98 mJ $cm^{-2}$), the width of the spaser line was drastically narrowing the spectral line to ~5 nm at 540 nm. In the experiments with the films, infiltrated by the dye only at the same concentration as in the spasers, only luminescence was observed at any pump intensity. Spasing radiation has a pronounced six-fold symmetry of the reciprocal space (the first Brillouin zone) (Supplementary Fig. 1C). The threshold is forty times lower and the amplification is much higher than for the same spasers in a suspension. Thus, these studies indicate a high potential of spherical spasers to enhance stimulated emission during clustering near optical surfaces. The planar spaser schematic provides opportunities for the development of biosensors and to study the interaction of normal and abnormal cells (e.g., circulating tumor cells) with surfaces.

**Supplementary Note 4 | Nanobubble spaser**

Laser-induced nanobubble phenomena are well described in the literature. In particular, we have pioneered the first applications of laser-induced vapor nanobubbles around plasmonic gold nanoparticles (NPs) and especially their self-assembled clusters in biological environment for effective killing of single cancer cells[5-22]. Vapor nanobubbles around clustered NPs significantly enhance the combination of PA diagnostics and photothermal (PT) therapy called PA-PT theranostics through synergy of thermal and mechanical cell destruction both tumors in static condition and circulating tumor cells in dynamic blood flow. After our pioneering demonstration of this technology in 2003-2005[5-9] using pulsed lasers, absorbing nanoclusters, and vapor nanobubbles, biomedical applications of this platform have become an extensive area of theoretical and experimental studies (e.g., see refs.23-31).

The mechanism of nanobubble formation is associated with PT heating of the absorbing NPs. When the surface temperature of NPs reaches the threshold for the evaporation of the liquid medium around them, it leads to formation of nanobubbles, whose expansion followed by a fast collapse enhances acoustic wave generation (referred as a PA signal). We discovered that these nonlinear phenomena are accompanied by dramatic (50-100-fold) narrowing ("sharpening") of PA spectra of plasmonic NPs and PA resonance splitting compared to linear NP absorption spectra[17]. The physical mechanism of these effects is based on the multistage PA signal behavior as energy fluence increases, particularly, a linear increase at low energy fluence and then a strong nonlinear nanobubble-related 10-50-fold signal amplification (Supplementary Fig. 6). A shift of the laser wavelength during spectral scanning toward the absorption center leads to increased energy absorption, raising the temperature above the nanobubble formation threshold and significantly amplifying PA signals near the absorption center. As a result, spectrally dependent signal amplification leads to the sharpening of PA (and PT) resonances only near the centers of absorption bands. At a higher laser energy, the

nonlinear signal amplification is changed to signal inhibition near the absorption center leading to spectral hole formation analogous to spectral "burning" of the center of absorption bands in conventional spectroscopy[17]. This phenomenon can occur due to several PT-related effects, including laser light scattering on a bubble at the beginning of the laser pulse. This is accompanied by a significant decrease in the absorbed energy and, hence, in the PA and PT signal amplitudes. However, the lower absorption outside the absorption center band still produces nanobubble-related PA signal amplification. In turn, this leads to the central ultrasharp PA peak splitting into two red- and blue-shifted sharp PA and PT spectral resonances (Fig. 2E)[17,20]. This spectral splitting phenomenon was also observed by another research group[30]. A significant asymmetry of the absorption band shape can lead to formation of one dominant frequently red-shifted resonance[17].

At a relatively low pump intensity, ≤ 20 MW $cm^{-2}$ (≤ 100 mJ $cm^{-2}$ for 5-10 ns laser pulse pump), a change in the spatial orientation of the slide with a spaser suspension with respect to the pump beam direction within an angle of ± 30° revealed almost isotropic emission distribution within the accuracy of 10-15 percent. This finding suggests an advantage of the spasers as optical probes because the signal is independent of the spatial orientation for the spasers inside cells with respect to both the direction of the pump laser and the axis of emission collection. At higher pump intensity, we observed interesting optical phenomena such as directional emission including emission focusing (Supplementary Fig. 9). These phenomena can be associated with the above-described formation of transient vapor nano- and microbubble (Fig. 1C, inset).

The size and lifetime of these bubbles, measured with various optical methods[5-30], were in the broad range of 30-50 nm to 10 µm and 20 ns to 5 µs, respectively, depending on pump intensity. Strong refractive, scattering and thermal lens effects in highly localized heated areas, especially in the associated bubbles can be responsible for the light concentrating and redirecting. We believe that these effects can be used to study nonlinear optical effects in the spaser and optimization of its biological applications using low pump intensity for diagnostics and an increased intensity for theranostics.

Nanobubble formation led to enhancement of spasing (Fig. 1C). The original (without a nanobubble) spaser is a nanoshell with a metal (Au) core covered with a dielectric shell containing dye molecules to produce the gain, embedded in the surrounding uniform medium, particularly, water (Fig. 1A). When a nanobubble is formed, another nanoshell appears between the gain shell and the embedding medium that contains water vapors.

To treat the problem analytically, we have employed the quasistatic approximation where the electric field potential in an *i*-th layer [*i*=1 (the metal core), *i*=2 (the gain shell), *i*=3 (the bubble shell), and *i*=4 (the embedding medium) is given by the Laplace equation $\nabla^2\phi_n(\mathbf{r})=0$. The boundary conditions at the surface of each layer (i.e., for $\mathbf{r}=\mathbf{r}_i$) are the standard Maxwell continuity conditions

$$\phi_i(\mathbf{r}_i)=\phi_{i+1}(\mathbf{r}_i)\,,\ \varepsilon_i\nabla_{\mathbf{n}}\phi_i(\mathbf{r})=\varepsilon_{i+1}\nabla_{\mathbf{n}}\phi_{n+1}(\mathbf{r})\big|_{\mathbf{r}=\mathbf{r}_i}\,, \tag{1}$$

where $\nabla_{\mathbf{n}}=\mathbf{n}\dfrac{\partial}{\partial r}$ is normal derivative to the surface at a point $\mathbf{r}$, and $\varepsilon_i$ is dielectric permittivity of the material in the corresponding region. The solution in the corresponding layers are given by

$$\phi_i(\mathbf{r})=\left(a_i r^{-2}+b_i r\right)Y_{10}(\mathbf{r})\,, \tag{2}$$

where $a_i, b_i$ are constant coefficients. These are found from boundary conditions, which result in a homogeneous linear system of equations,

$$b_1 r_1^3 = a_2 + b_2 r_1^3,\ a_2 + b_2 r_2^3 = a_3 + b_3 r_2^3,\ a_3 + b_3 r_3^3 = a_4 + b_4 r_3^3, \\ -\varepsilon_1 b_1 r_1^3 = \varepsilon_2 \left(2a_2 - b_3 r_1^3\right),\ \varepsilon_2 \left(2a_2 - b_2 r_2^3\right) = \varepsilon_3 \left(2a_3 - b_3 r_2^3\right), \\ \varepsilon_3 \left(2a_3 - b_3 r_3^3\right) = \varepsilon_4 \left(2a_4 - b_4 r_3^3\right) \tag{3}$$

A solution for homogeneous system Eq. (3) is generally trivial (zero) except if its determinant vanishes. A condition of this is a certain relation between its parameters. This can be expressed as an eigenvalue of the system, $\varepsilon_1$ expressed as a certain function of the other parameters, $\varepsilon_1 = \varepsilon_1\left(\varepsilon_2, \varepsilon_3, \varepsilon_4, r_1, r_2, r_3\right)$. A physical frequency of a plasmonic eigenmode, $\omega_n$, is found from an equation $\varepsilon_{\rm Au}(\omega_n) = \varepsilon_1$, where $\varepsilon_{\rm Au}$ is an experimentally known[31] permittivity of gold.

Further analytical computations were conducted exactly using Mathematica 11. The corresponding results are too bulky to be reproduced in print but are available as a Mathematica file upon request. To describe spaser operation based on these eigenmodes, we generally followed the previously published quantum-mechanical theory[32]. Full details of this theory for the nanobubble spaser will be published elsewhere.

The main effect of the formation of a vapor nanobubble around the spaser is that the dielectric screening of the surface plasmon-induced charges is reduced. This leads to an increase of the surface plasmon frequency, $\omega_n$, as illustrated in Supplementary Fig. 8. In fact, the spaser frequency increases from the initial value of $\omega_n = 2.5$ eV in the absence of the nanobubble to $\omega_n = 2.6$ eV for a 30-nm radius nanobubble (thickness of the nanobubble shell of $r_3 - r_2 = 18$ nm).

If the gain-medium working (spasing) transition frequency, $\omega_{21}$, exceeds the plasmon frequency, then the nanobubble formation brings the spaser closer to a perfect resonant condition. Consequently, the stimulated radiation of the surface plasmons becomes more efficient, which is the case in the present article; the corresponding theoretical result is shown in Fig. 1D. This explains the “giant spasing” effect observed in the article (Fig. 1C).

It is important to note that the nanobubbles can provide dynamic optical feedback from its “wall” boundary, as well as refraction effects that can also lead even to directional spaser emission (Supplementary Fig. 9).

**Supplementary Note 5 | Spaser assessment with inductively coupled plasma mass spectrometry (ICP-MS).**

In order to quantify the uptake of spasers by cells, elemental analysis was performed using inductively coupled plasma mass spectrometry (ICP-MS). Samples were digested with HCL and HF (1:3) in an SPD microwave. The calibration with controlled samples demonstrated a linear dependence of the signal intensity on the Au concentration, MDL = 0.6345 and spiking recovery of 104.52%. A full quant ICO-MS revealed the presence of Au in samples of spasers with cells at different incubation time that is in line with data obtained with PA flow cytometry (Supplementary, Fig.12, Table 1).

**Supplementary Note 6 | Spaser assessment by integrated STEM and EDX techniques.**

Scanning transmission electron microscopy (STEM) with energy-dispersive x-ray spectroscopy (EDX) was performed with JEOL JEM 2100F (JEOL USA, Peabody, MA) equipped with an EDAX Genesis (Ametek, Berwyn, PA) EDX x-ray analyzer. The obtained data (Supplementary Fig. 3,11) shows the presence Au, Si, and O from spasers, as well as elements from the substrate the STEM grid. C is present from the carbon films on STEM grid, but also due to the biological agents present in the solution that the spasers were dissolved in.

**Supplementary Note 7 | Spaser photodamage**

Measurement of fluorescent intensity from the spasers and the dye alone in solution and in gel at different pump laser fluences indicated no photobleaching at a relatively high fluence of 0.06-0.1 J $cm^{-2}$. The spasers were more resistant to photobleaching than the dye alone suggesting that the silica matrix stabilizes the dye and the faster transfer of energy from the dye to the spaser core protects it from degradation. In gel with more restricted spatial motion, the photobleaching was a little more profound (Supplementary Fig. 14). Spaser-produced emission intensity inside cells (Fig. 2C) was somewhat lower compared to emission in suspension, as well as spectrally wider (3-5 nm) sometimes containing a few (2-4) closely located peaks with total width of 5-15 nm due to lower spaser concentration inside the cells than in suspension before incubation with the cells. At low pump energy, the cells remained alive even after prolonged laser exposure up to 1,000 pulses at 100 nJ/pulse, which implies an optically-nonlinear nature of the photodamage.

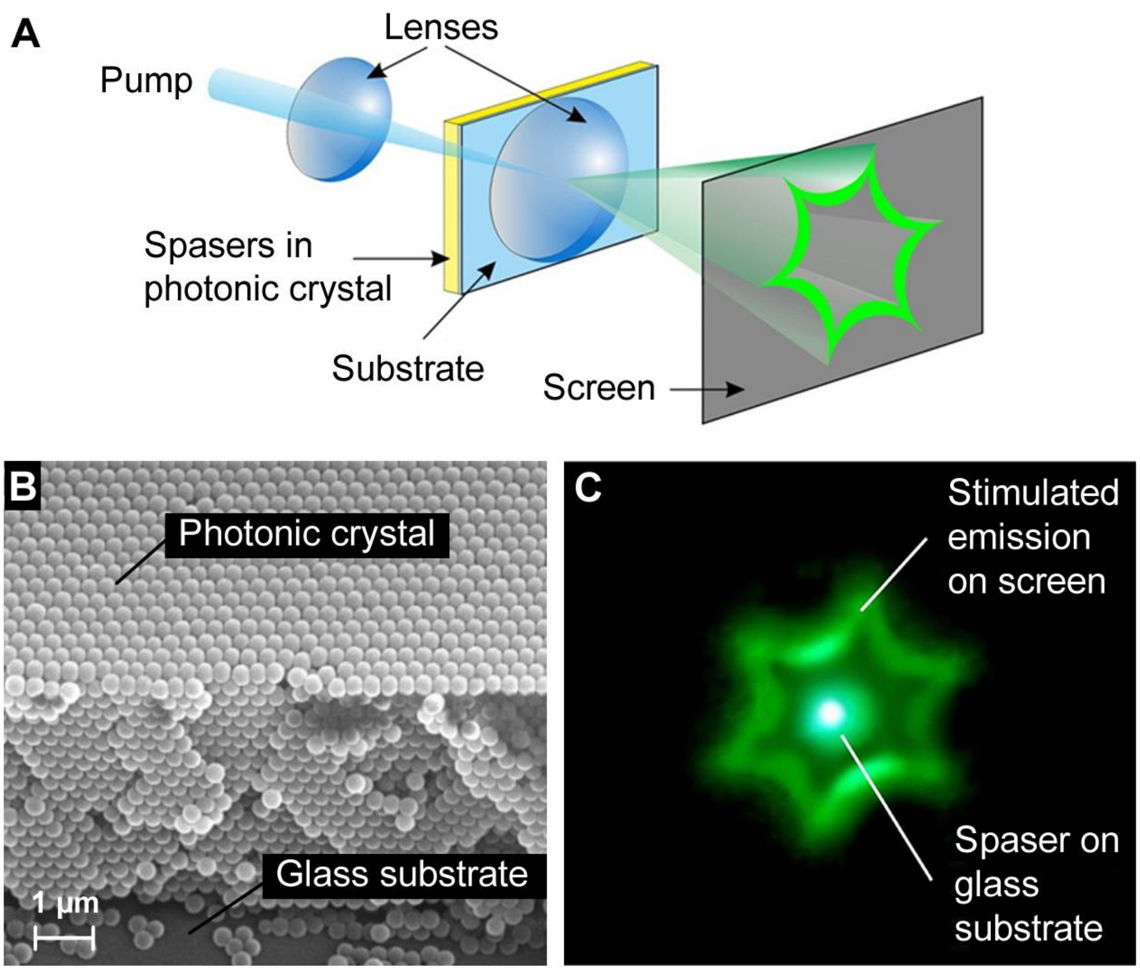


**Supplementary Figure 1 | Film-based spasers**. (**A**) Schematic. The pump parameters: wavelength, 488 nm; pulse width, 7 ns. (**B**) Top view of the edge of the synthetic film. (**C**) Photo of the distribution of lasing spaser radiation in the far field in the form of a six-pointed star[3,4].

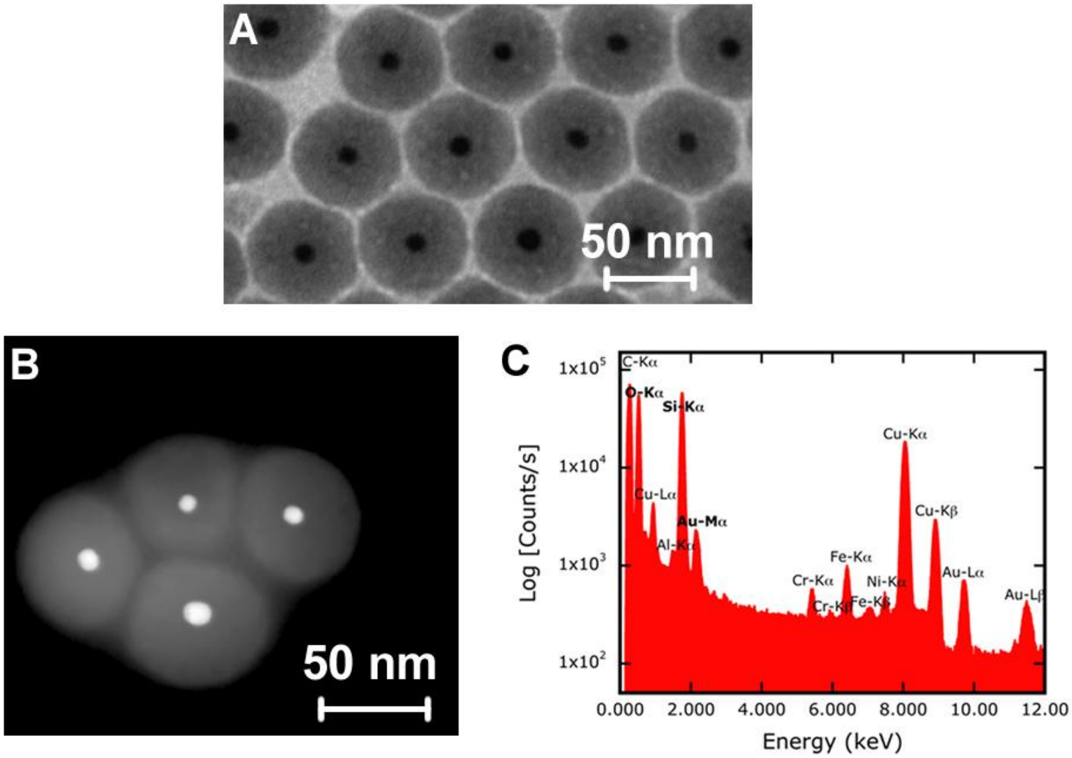


**Supplementary Figure 2 | Imaging of spasers**. (**A**) TEM images of 60 nm spasers showing gold core (central dark spot) and surrounding silica layer (grey shell). (**B**) STEM dark-field images of spasers showing bright gold cores and silica shells. (**C**) EDX spectrum of spasers collected during the STEM analysis. It shows the presence of Si, O, and Au. Since the Au volumes in spasers are relatively small compared to that of $SiO_2$ shell, Au-L and Au-M peaks are relatively small compared to Si-K and O-K (as well as C-K, Cu-K and Cu-L peaks from the grid). Cr, Fe and Ni are also impurities present on the TEM grid. O-K, Si-K and Au-M peaks are presented in the STEM/EDX elemental maps (below) and are labeled in bold letters here.

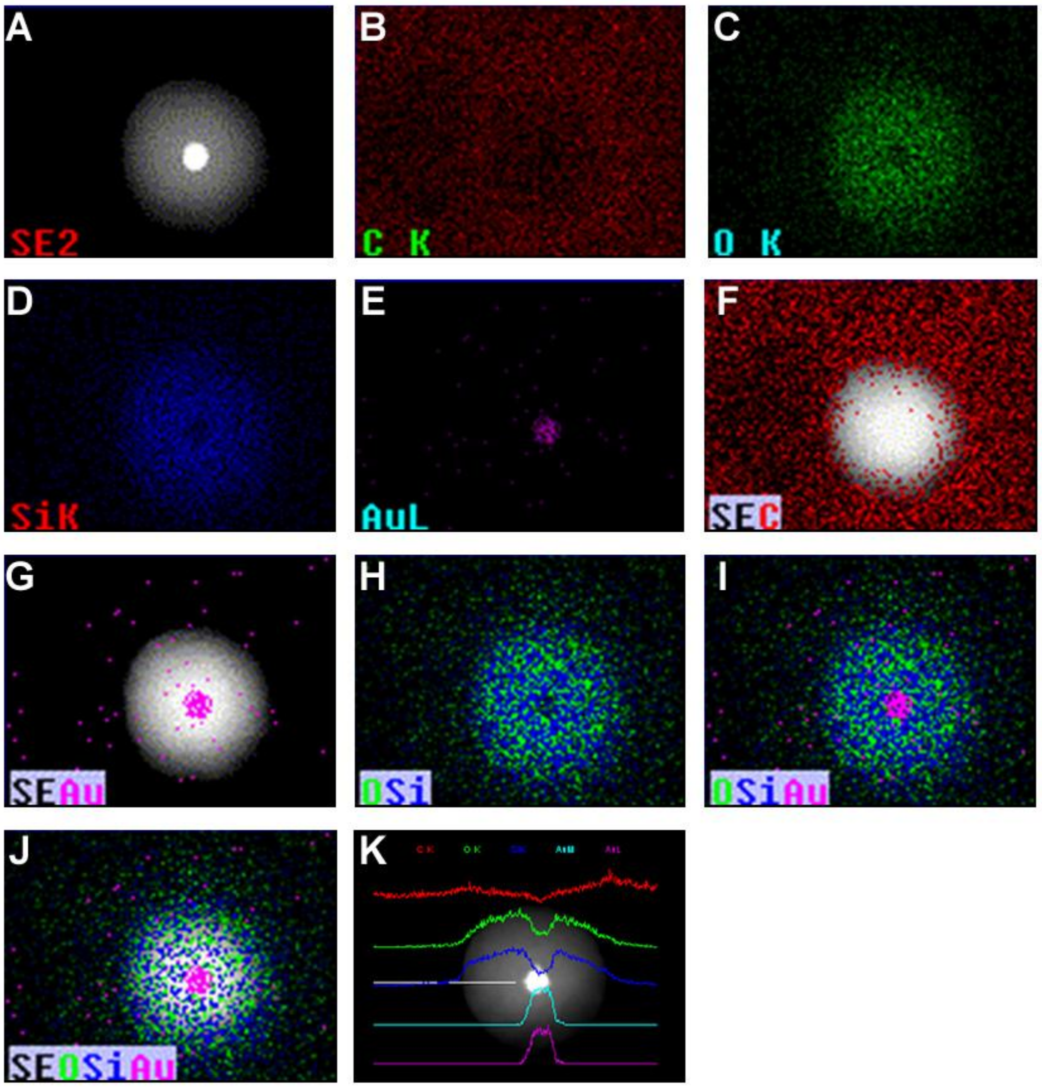


**Supplementary Figure 3 | Integration of imaging and elemental maps of a single spaser.** (**A**) STEM dark-field electron image taken with the x-ray maps. (**B**) C-K x-ray map. (**C**) O-K x-ray map. (**D**) Si-K x-ray map. (**E**) Au-L x-ray map. (**F**) Overlay of electron image with C-K X-ray (**A** and **B**). (**G**) Overlay of electron image with Au-L x-ray (**A** and **E**). (**H**) Overlay of O-K x-ray and Si-K x-ray (**C** and **D**). (**I**) Overlay of O-K x-ray, Si-K x-ray, and Au-L x-ray (**C**, **D**, and **E**). (**J**) Overlay of electron image with O-K x-ray, Si-K x-ray, and Au-M x-ray (**A**, with **C**, **D**, and **E**). (**K**) Elemental line scans across the center of this spaser (red: C-K, green: O-K, blue: Si-K, magenta: Au-L, and purple: Au-M intensities).

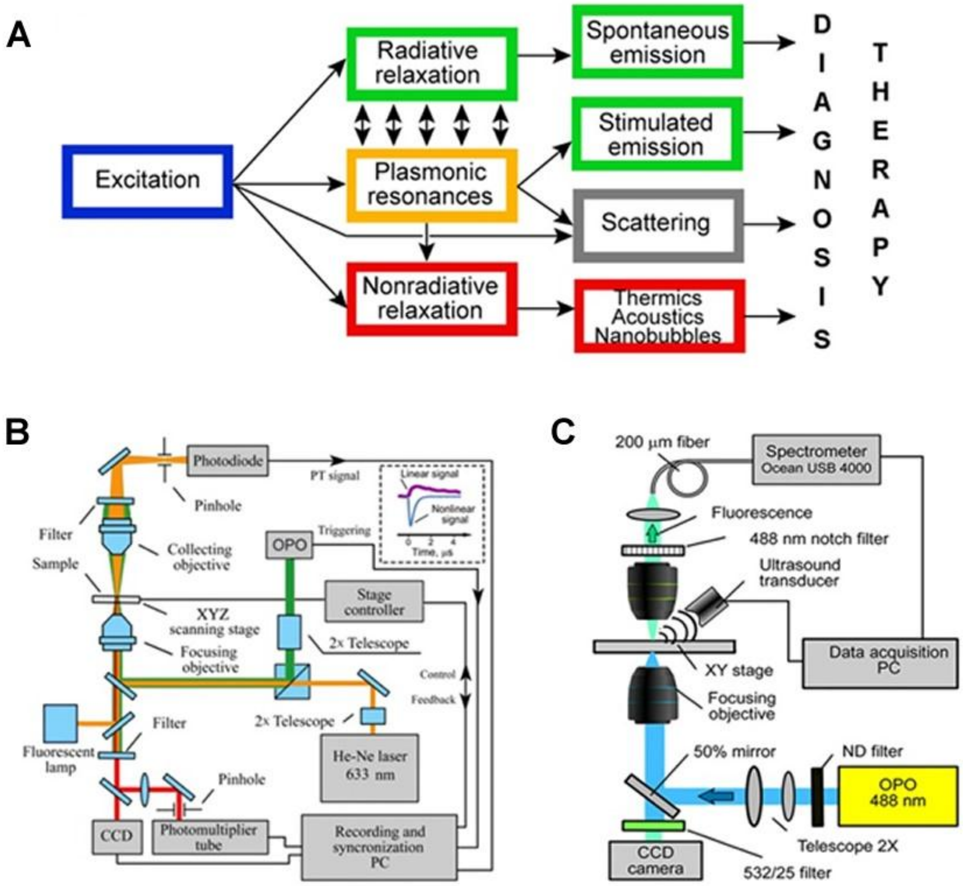


**Supplementary Figure 4 | Schematics of experiments setups**[5-7]. (**A**) Basic physical phenomena under laser-spaser interaction with focus on spontaneous and stimulated emission and photothermal (PT) and photoacoustic (PA) effects. (**B**) Integrated PT, PA, and fluorescence microscopy. (**C**) Integrated PA and fluorescence microscopy and spectroscopy using Ocean USB 4000 spectrometer.

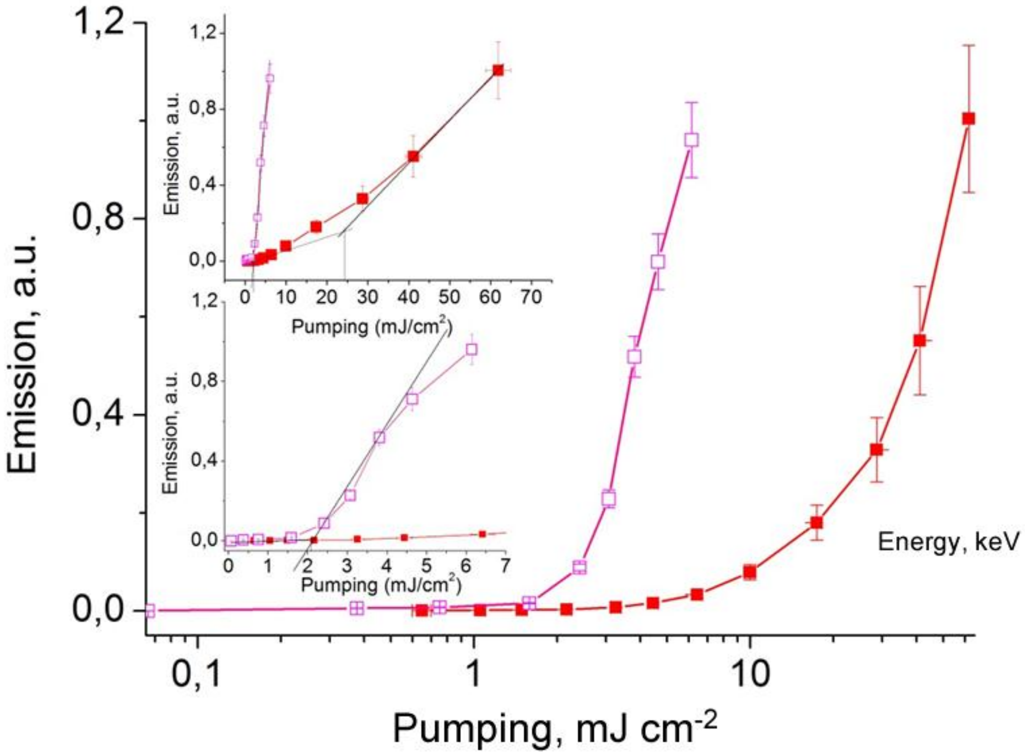


**Supplementary Figure 5 | Spaser emission.** The emission intensity as a function of pump energy (input-output curve) for spasers with a diameter of 22 nm (red filled squares) and 60 nm (pink empty squares). Two curves are normalized to energy of 1 mJ cm$^{-2}$ and presented in a logarithmic scale on the horizontal axis for ease of comparison. The insets provide more details using linear scales. These data showed the threshold fluences of stimulated emission around 26 ± 6.3 mJ cm$^{-2}$ and 1.9 ± 0.6 mJ cm$^{-2}$ for spasers with a diameter of 22 nm, and 60 nm, respectively. Standard deviations (SD) for intensity and the threshold are in the range of 18-26%.

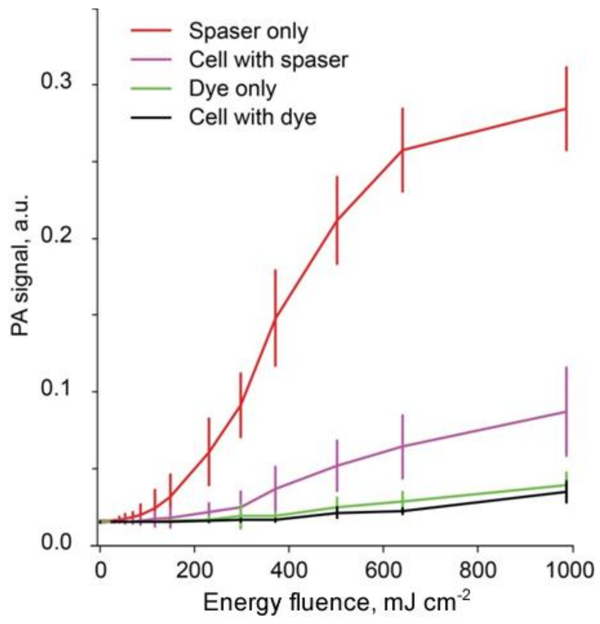


**Supplementary Figure 6 | The dependence of PA signal amplitude on laser energy fluence for dye and spasers in suspension and in cells**. Comparison of the PA signal amplitude from the dye and the spasers in water and in cells reveals nanobubble-associated nonlinear signal increase from the spaser core at 532 nm alone and inside cells.

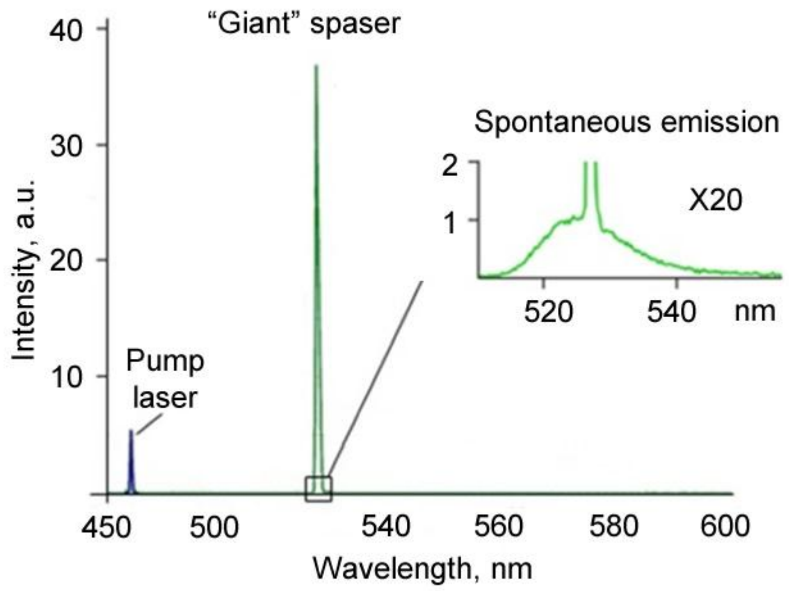


**Supplementary Figure 7 | Giant spaser**. Radiation spectrum of 60-nm spaser in suspension near 528 nm at pump energy fluence of 250 mJ $cm^{-2}$ (green). The inset shows spontaneous emission background. Attenuated intensity of pump laser is shown in blue.

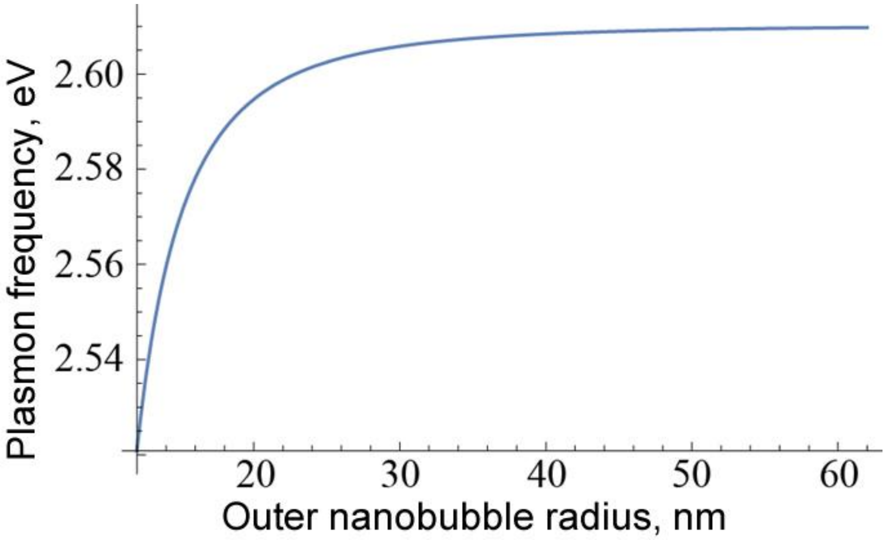


**Supplementary Figure 8 | Theoretical modeling**. Theoretical dependence of the spaser mode frequency, $\omega_n$, on the outer radius of the bubble, $r_3$. Parameters used in the computations are: $r_1 = 7$ nm, $r_2 = 12$ nm; $\varepsilon_2 = 6$, $\varepsilon_3 = 1$ (nanobubble), $\varepsilon_4 = 1.8$; $\omega_{21} = 2.87$ eV.

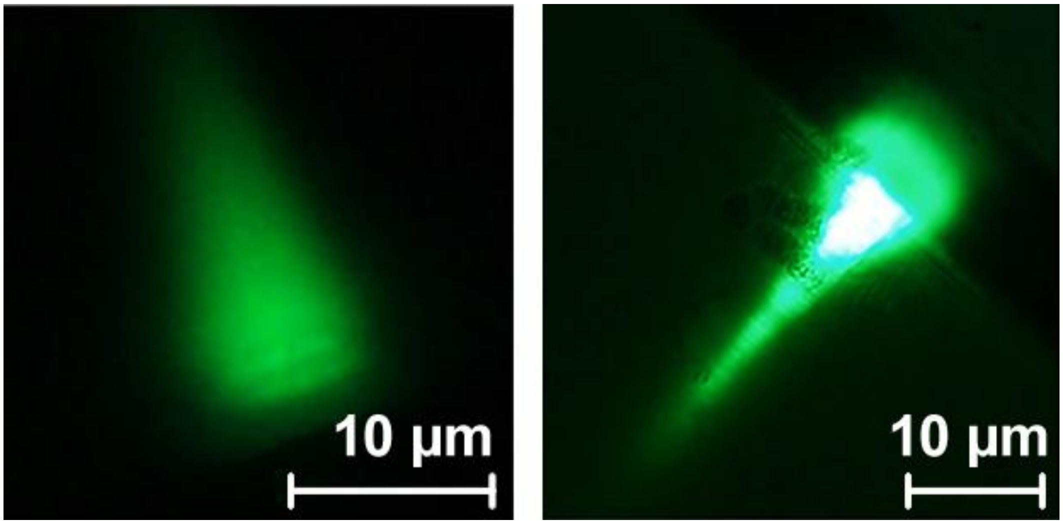


**Supplementary Figure 9 | Directional spaser emission**. Fluorescence images of nanobubble- and microbubble -induced directional emission of spasers in solution in 120-µm thick slide under exposure by pump pulse with diameter of 10 µm, wavelength of 488 nm, and energy fluence of 80 mJ cm$^{-2}$ (**left**) and 130 mJ cm$^{-2}$ (**right**).

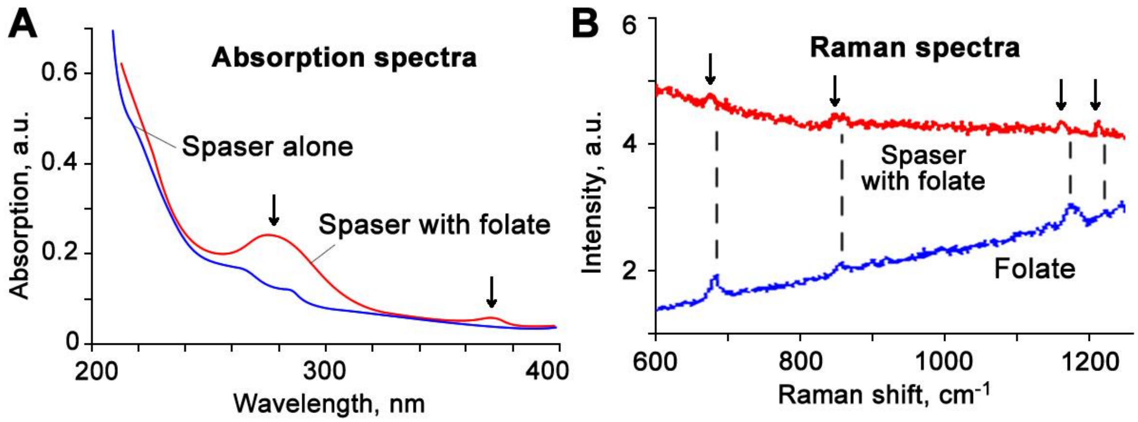


**Supplementary Figure 10 | Spectral verification of conjugation of spaser with folic acid.** (**A**) Absorption spectra of spasers alone and spasers with folic acid showing the peaks at 280 nm and 371 nm (arrows) specific for folic acid. (**B**) Raman spectra of folate alone and spasers with folate showing coincidence of specific peaks with slight blue shifting associated likely with the influence of the spaser shell.

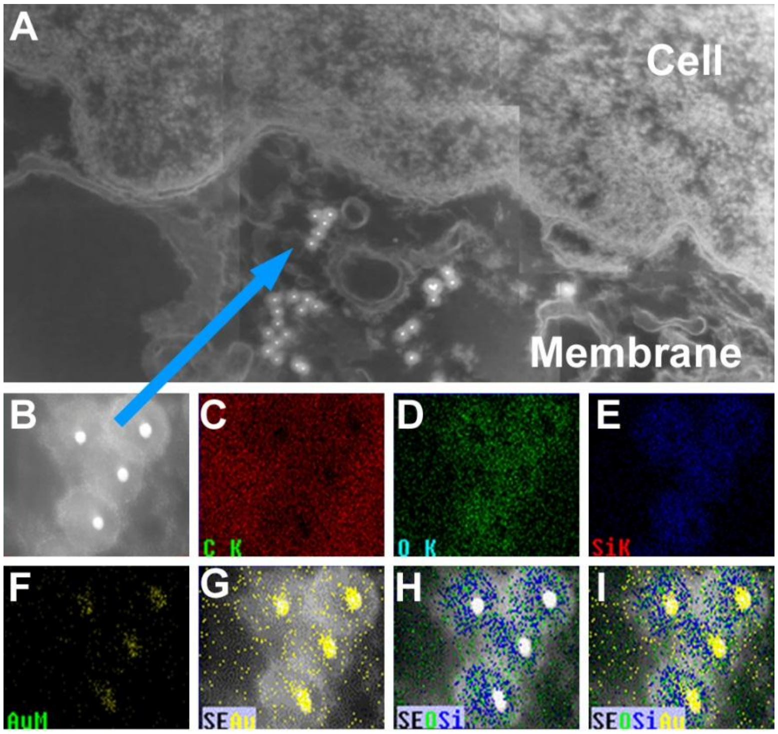


**Supplementary Figure 11 | Integration of imaging and elemental mapping of spasers into cancer cells**. **(A)** Composite of low magnification STEM dark field images spaser nanoparticles near a cell; (**B)** STEM dark field image taken with the x-ray maps. (**C)** C-K x-ray map. (**D)** O-K x-ray map. (**E)** Si-K x-ray map**. (F)** Au-M x-ray map. (**G)** Overlay of dark field image with Au-M x-ray map (**B** and **F)**. (**H)** Overlay of dark field image with O-K x-ray map and Si-K x-ray map (**B, D,** and **E)**. (**I)** Overlay of dark field image, O-K x-ray map, Si-K x-ray map, and Au-M x-ray map (**B, D**, **E,** and **F)**. The elements with higher atomic numbers appear brighter in dark field images, while the elements with lower atomic numbers appear dark in bright field images (the contrast is reversed).

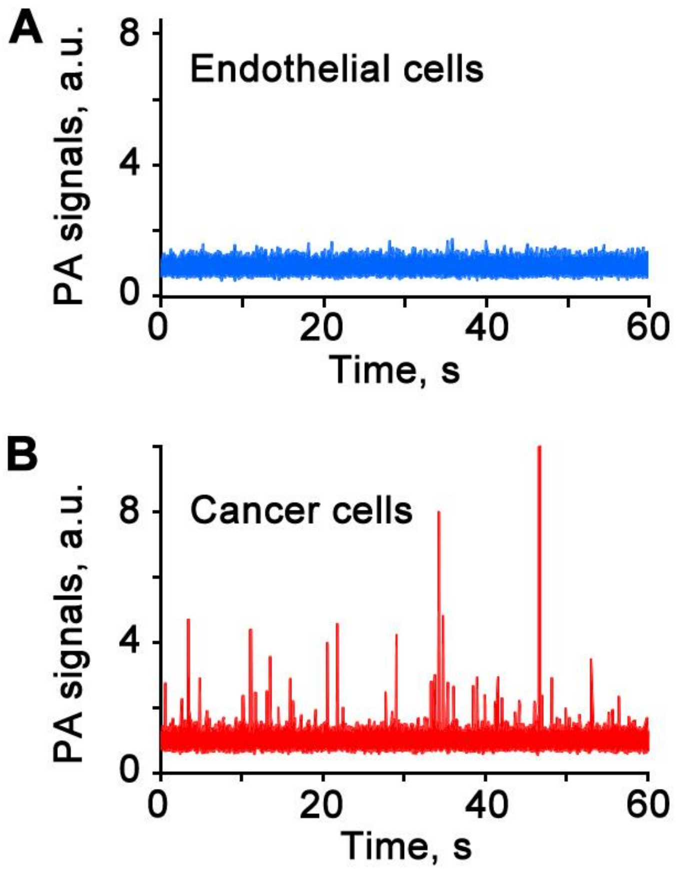


**Supplementary Figure 12 | PA flow cytometry[6,7] of labeled cells *in vitro***. PA signal traces of endothelial (**A**) and cancer (MDA-MB-231) cells (**B**) targeted by folic acid-conjugated spasers.

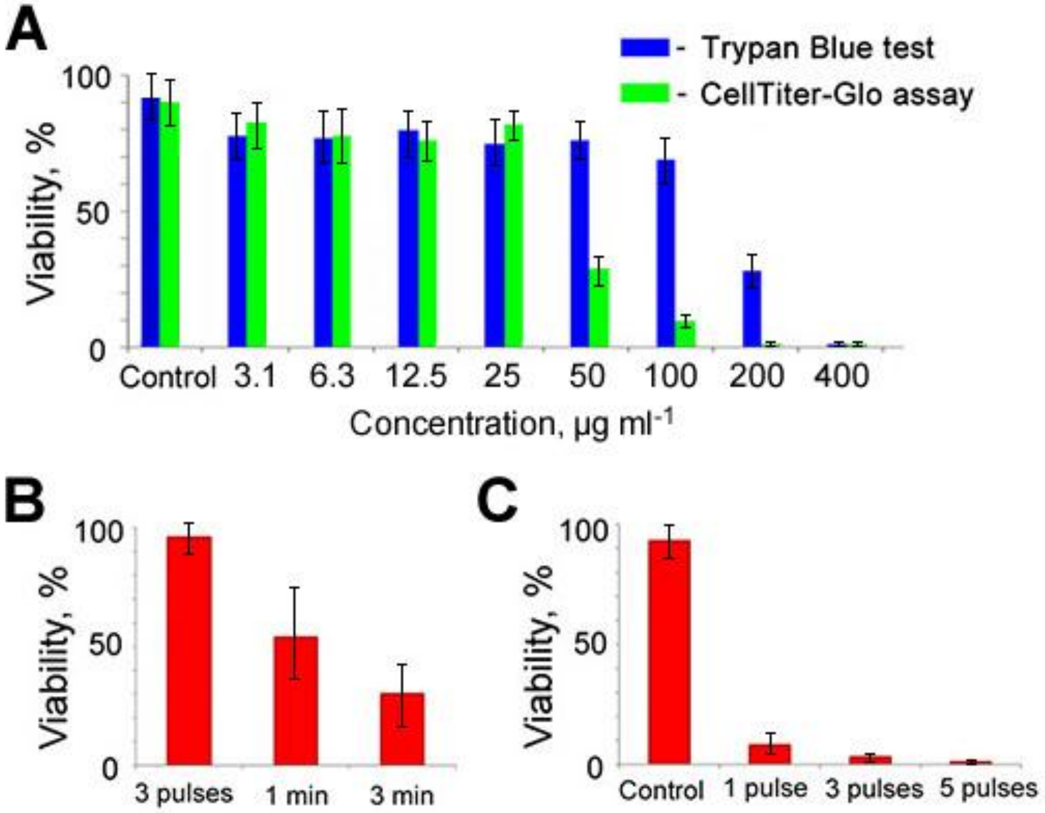


**Supplementary Figure 13 | Toxicity study**. **(A)** Cell viability tests for different spaser concentration using two various kits: Trypan Blue and Cell Titer-Glot. (**B)** Viability (Trypan Blue) test for the 3.1 µg ml$^{-1}$ concentration as a function of laser exposure time (3s [3 pulses], 1 min, and 3 min) at pulse rate of 1 Hz; (**C)** Viability (Trypan Blue) test for the 3.1 µg ml$^{-1}$ concentration as a function of laser pulse number (1, 3 and 5). It shows that even single laser pulse with a fluence of 500 mJ cm$^{-2}$ is sufficient for significant damage of cancer cells labeled by spasers. The average SD for each column is 15-20%.

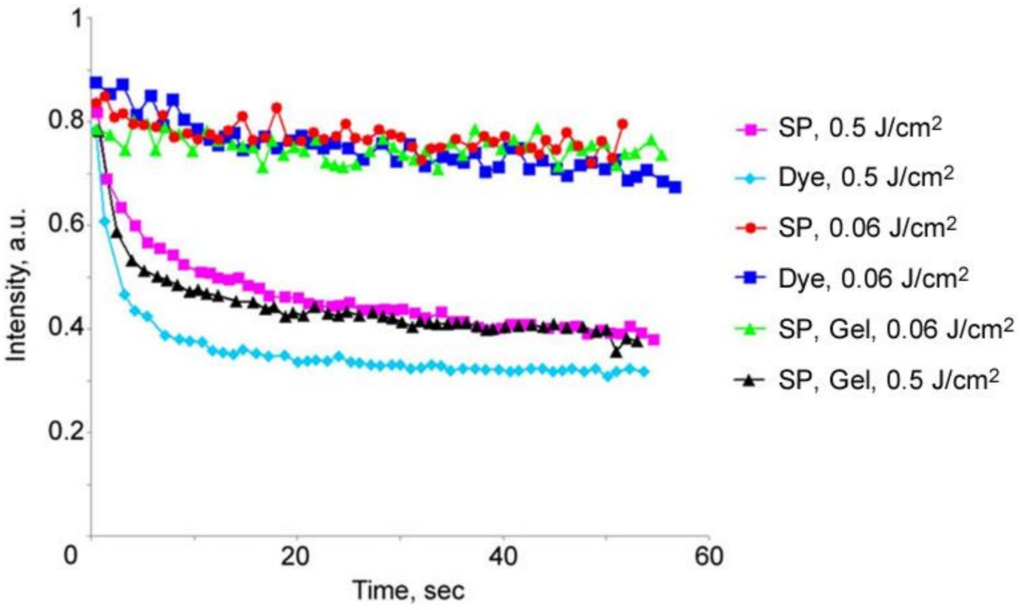


**Supplementary Figure 14 | Spaser photobleaching**. Fluorescence intensity from spasers (SP) and dye alone at high (0.5 J $cm^{-2}$) and low (0.06 J $cm^{-2}$) pump laser energy fluence as a function of exposure time at a laser pulse rate of 10 Hz in suspension and in gel. The average SD for each time point is 16%.

**Supplementary Table 1. Control of spasers in breast cancer cells (MDA-MB-231) with ICP-MS**

| Sample | Concentration of Au (ppb) | SD |
|---|---|---|
| Cancer cells, control | 0 | 0.69 |
| Cancer cells incubated with spaser for 30 min, 37 °C | 34.61 (62%) | 0.25 |
| Cancer cells incubated with spaser for 60 min, 37 °C | 42.30 (79%) | 0.70 |
| Spaser alone (calibration) | 55.14 (100%) | 1.25 |

## Supplementary References


1. Meng, X. G., Kildishev, A. V., Fujita, K., Tanaka, K. & Shalaev, V. M. Wavelength-tunable spasing in the visible. *Nano Letters* **13,** 4106-4112 (2013).
2. Benimetskiy, F., Plekhanov, A., Kuchyanov, A., Parkhomenko, R. & Basova, T. Characterization of the structure and stimulated emission of spherical and cylindrical spasers. in *Days on Diffraction* **2016,** 62-66 (St. Petersburg, Russia, 2016).
3. Parkhomenko, R. G. *et al.* Gold nanostructure formation in the photonic crystal matrix by means of MOCVD technique. *Surface & Coatings Technology* **230,** 279-283 (2013).
4. Kuchaynov, A. S. *et al*. External-cavity spasers. in *The Seventh International Conference on Material Technologies and Modeling MMT-2012*, 67–71 (Ariel, Israel, 2012).
5. Zharov, V.P., Galitovsky, V. & Vegas, M. Photothermal detection of local thermal effects during selective nanophotothermolysis. *Appl Phys Letter* **83,** 4897-4899 (2003).
6. Zharov, V.P., Galitovskaya, E.N. & Viegas, M. Photothermal guidance for selective photothermolysis with nanoparticles. Proc. SPIE **5319** (2004).
7. Zharov, V.P., Galitovskaya, E.N., Johnson, C. & Kelly, T. Synergistic enhancement of selective photothermolysis with gold nanoclusters: potential for cancer therapy. *LaserSurg Med* **37,** 219-226 (2005).
8. Zharov, V.P., Letfullin, R.R. & Galitovskaya, E.N. Microbubbles-overlapping mode for laser killing of cancer cells with absorbing nanoparticle clusters. *J. Phys. D: Appl. Phys*. **38,** 2571-2581 (2005).
9. Zharov, V.P., Kim, J.W., Curiel, D.T. & Everts, M. Self-assembling nanoclusters in living systems: application for integrated photothermal nanodiagnostics and nanotherapy. *Nanomedicine* **1,** 326-345 (2005).
10. Khlebtsov, B., Zharov, V.P., Melnikov, A., Tuchin, V. & Khlebtsov, N. Optical amplification of photothermal therapy with gold nanoparticles and nanoclusters. *Nanotechnology* **17,** 5167-5179 (2006).

11. Letfullin, R.R., Joenathan, C., George, T.F. & Zharov, V.P. Laser-induced explosion of gold nanoparticles: potential role for nanophotothermolysis of cancer. *Nanomedicine (Lond)* **1,** 473-480 (2006).
12. Pustovalov, V.K., Smetannikov, A.S. & Zharov, V.P. Photothermal and accompanied phenomena of selective nanophotothermolysis with gold nanoparticles and laser pulses. *Laser Phys. Lett*. **5,** 775-792 (2008).
13. Shashkov, E.V., Everts, M., Galanzha, E.I. & Zharov, V.P. Quantum dots as multimodal photoacoustic and photothermal contrast agents. *Nano Lett*. **8,** 3953-3958 (2008).
14. Akchurin, G. et al. Gold nanoshell photomodification under a single-nanosecond laser pulse accompanied by color-shifting and bubble formation phenomena. *Nanotechnology* **19,** 015701 (2008).
15. Galanzha, E. I. et al. In vivo magnetic enrichment and multiplex photoacoustic detection of circulating tumour cells. *Nature Nanotechnology* **4,** 855-860 (2009).
16. Kim, J.W., Galanzha, E.I., Shashkov, E.V., Moon, H. M. & Zharov, V.P. Golden carbon nanotubes as multimodal photoacoustic and photothermal high-contrast molecular agents. *Nat Nanotechnol*. **4,** 688-694 (2009).
17. Zharov, V. P. Ultrasharp nonlinear photothermal and photoacoustic resonances and holes beyond the spectral limit. *Nature Photonics* **5,** 110-116 (2011).
18. de la Zerda, A., Kim, J.W., Galanzha, E.I., Gambhir, S.S. & Zharov, V.P. Advanced contrast nanoagents for photoacoustic molecular imaging, cytometry, blood test and photothermal theranostics. *Contrast Media Mol Imaging* **6,** 346-369 (2011).
19. Kim, J.W., Galanzha, E.I., Zaharoff, D.A., Griffin, R.J. & Zharov, V.P. Nanotheranostics of circulating tumor cells, infections and other pathological features *in vivo*. *Mol Pharm*. **10,** 813-830 (2013).
20. Shao, J. et al. Photothermal nanodrugs: potential of TNF-gold nanospheres for cancer theranostics. *Sci Rep*. **3**:1293 (2013).
21. Galanzha, E.I. & Zharov, V.P. Circulating tumor cell detection and capture by photoacoustic flow cytometry in vivo and *ex vivo. Cancers (Basel)* **5,** 1691–1738 (2013).
22. Sarimollaoglu, M., Nedosekin, D. A., Menyaev, Y. A., Juratli, M. A. & Zharov, V. P. Nonlinear photoacoustic signal amplification from single targets in absorption background. *Photoacoustics* **2,** 1-11 (2014).
23. Wen, D. Intracellular hyperthermia: Nanobubbles and their biomedical applications. *Int J Hyperthermia* **25,** 533-541 (2009).
24. González, M.G., Liu, X., Niessner. R. & Haischa, C. Strong size-dependent photoacoustic effect on gold nanoparticles by laser-induced nanobubbles. *Appl Phys Letter* **96,** 174104 (2010).

25. Siems, A., Weber, S.A.L., Boneberg, J. & Plech A. Thermodynamics of nanosecond nanobubble formation at laser-excited metal nanoparticles. *New Journal of Physics* **13,** 043018 (2011).
26. Lukianova-Hleb, E.Y. et al. Tunable plasmonic nanoprobes for theranostics of prostate cancer. *Theranostics* **1**: 3-17 (2011).
27. Furlani, E.P., Karampelas, I.H. & Xie, Q. Analysis of pulsed laser plasmon-assisted photothermal heating and bubble generation at the nanoscale. *Lab Chip* **12,** 3707-3719 (2012).
28. Lukianova-Hleb E.A. & Lapotko, D.O. Experimental techniques for imaging and measuring transient vapor nanobubbles. *Appl. Phys. Lett.* **101,** 264102 (2012).
29. Lombard, J., Biben, T. & Merabia, S. Kinetics of nanobubble generation around overheated nanoparticles. *Phys. Rev. Lett*. **112,** 105701 (2014).
30. Mertiri, A. et al. Nonlinear midinfrared photothermal spectroscopy using Zharov splitting and quantum cascade lasers. *Acs Photonics* **1,** 696-702 (2014).
31. Johnson, P. B. & Christy, R. W. Optical constants of the noble metals. *Physical Review B* **6,** 4370-4379 (1972).
32. Stockman, M. I. The spaser as a nanoscale quantum generator and ultrafast amplifier. *Journal of Optics* **12,** 024004 (2010).
